# Supplementary material for: Efficient and practical Hamiltonian simulation from time-dependent product formulas
Source: Nat Commun. 2025 Mar 26;16:2673. doi: 10.1038/s41467-025-57580-5 (PMC11947201; doi:10.1038/s41467-025-57580-5)
Supplement: Supplementary file 1 — Supplementary Information [file 41467_2025_57580_MOESM1_ESM.pdf]

Supplementary Information for "Efficient and practical Hamiltonian simulation from time-dependent product formulas"

Jan Lukas Bosse[1,2], Andrew M. Childs[1,3], Charles Derby[1], Filippo Maria Gambetta[1], Ashley Montanaro[1,2], and Raul A. Santos[1]

[1]Phasecraft Ltd.
[2]School of Mathematics, University of Bristol
[3]Department of Computer Science, Institute for Advanced Computer Studies, and Joint Center for Quantum Information and Computer Science, University of Maryland

February 20, 2025

Supplementary Note 1 – Error scaling of THRIFT

1 Commutator scaling

THRIFT methods approximate an interaction picture evolution unitary to pth order in t via a time-dependent product formula of the form

$$S_p(t) = e^{H_0 t} \prod_{v=1}^{\Upsilon} \prod_{\gamma=1}^{\Gamma} \mathcal{T} e^{\int_{a_{v-1}t}^{a_v t} H_{\pi_v(\gamma)}(s) ds}. \tag{1}$$

Note that, to reduce clutter and to avoid keeping track of phases, the factors of i are absorbed into the Hamiltonians in the following analysis. The results are unaffected by this choice.

Using the fact that the time dependence of the $H_\gamma(t)$ is simply unitary evolution under H_0, this is converted back to an equivalent product formula of time-independent terms

$$S_p(t) = \prod_{v=1}^{\Upsilon} e^{(H_{\pi_v(\Gamma)}+H_0)A_v t} e^{-H_0 A_v t} e^{(H_{\pi_v(\Gamma-1)}+H_0)A_v t} e^{-H_0 A_v t} \cdots e^{-H_0 A_v t} e^{(H_{\pi_v(1)}+H_0)A_v t}, \tag{2}$$

where $A_v = a_v - a_{v-1}$ with $a_0 = 0$. This is essentially a Trotter-style product formula of the Hamiltonian $H = H_0 + \sum_{\gamma=1}^{\Gamma} H_\gamma$ where H is decomposed into the sum

$$H = \sum_{l=1}^{2\Gamma-1} \tilde{H}_l \tag{3}$$

with $\tilde{H}_{2\gamma-1} = H_\gamma + H_0$ and $\tilde{H}_{2\gamma} = -H_0$. This product formula fits the general form used in [1],

$$S_p(t) = \prod_{v=1}^{\Upsilon} \prod_{l=1}^{2\Gamma-1} e^{\tilde{H}_{\pi_v(l)} A_v t}, \tag{4}$$

and by the main result of that work, the additive and multiplicative errors $\mathcal{A}(t)$, $\mathcal{M}(t)$, defined as

$$S_p(t) = e^{Ht} + \mathcal{A}(t) = e^{Ht}(I + \mathcal{M}(t)), \tag{5}$$

both scale as

$$\|\mathcal{A}(t)\|, \|\mathcal{M}(t)\| = O(\tilde{T}_p t^{p+1}), \tag{6}$$

where

$$\tilde{T}_p = \sum_{l_1,\ldots,l_{p+1}=1}^{2\Gamma-1} \|[\tilde{H}_{l_{p+1}}, \cdots [\tilde{H}_{l_2}, \tilde{H}_{l_1}]\cdots]\|, \tag{7}$$

with $\|\cdot\|$ denoting spectral norm. Expanding commutators containing terms of the form $H_\gamma + H_0$ and applying the triangle inequality, we have

$$\tilde{T}_p \leq \sum_{\gamma_1,\ldots,\gamma_{p+1}=0}^{\Gamma} C_{\gamma_1,\ldots,\gamma_{p+1}} \|[H_{\gamma_{p+1}}, \cdots [H_{\gamma_2}, H_{\gamma_1}]\cdots]\|, \tag{8}$$

where the sum is now over $\{H_0, \ldots, H_\Gamma\}$ and $C_{\gamma_1,\ldots,\gamma_{p+1}}$ are constants. Setting $C_{\Gamma,p} = \max\{C_{\gamma_1,\ldots,\gamma_{p+1}}\}$ (dependent only on Γ and p) and defining

$$T_p = \sum_{\gamma_1,\ldots,\gamma_{p+1}=0}^{\Gamma} \|[H_{\gamma_{p+1}}, \cdots [H_{\gamma_2}, H_{\gamma_1}]\cdots]\|, \tag{9}$$

we have

$$\tilde{T}_p \leq C_{\Gamma,p} T_p. \tag{10}$$

Taking Γ and p as constant, we then have for a pth-order THRIFT product formula

$$\|\mathcal{A}(t)\|, \|\mathcal{M}(t)\| = O(T_p t^{p+1}), \tag{11}$$

i.e., the same asymptotic scaling as a standard product formula for the decomposition into $\{H_0, \ldots, H_\Gamma\}$.

A commutator treatment for average-case product formula error is given in [2]. They show that for a pth-order product formula approximating evolution under $\sum_{\gamma=0}^{\Gamma} H_\gamma$ applied to states drawn from a 1-design input ensemble, the average error in the l_2 norm is bounded asymptotically as

$$R_{l_2} = O(T_p^F t^{p+1}), \tag{12}$$

where

$$T_p^F = \sum_{\gamma_1,\ldots,\gamma_{p+1}=0}^{\Gamma} \frac{1}{\sqrt{d}} \|[H_{\gamma_{p+1}}, \cdots [H_{\gamma_2}, H_{\gamma_1}]\cdots]\|_F, \tag{13}$$

with $\|H\|_F = \sqrt{\mathrm{Tr}[HH^\dagger]} \leq \sqrt{d}\|H\|$ denoting the Frobenius norm. By the same argument as above for the spectral error, this asymptotic bound applies equally to THRIFT.

2 System size scaling for geometrically local Hamiltonians

Given a d-dimensional lattice Λ^d of n qubits with distance metric D, define a geometrically local Hamiltonian as

$$H = \sum_{Z \subset \Lambda^d} H_Z \tag{14}$$

where H_Z acts only on a finite subset of lattice sites Z and there exists a constant, finite R such that

$$\|H_Z\| \leq \begin{cases} 1 & \text{if } \operatorname{diam}(Z) \leq R \\ 0 & \text{if } \operatorname{diam}(Z) > R, \end{cases} \tag{15}$$

where $\operatorname{diam}(Z) = \max\{D(i,j) : i, j \in Z\}$ is the maximum distance between any two points in Z.

Supplementary Lemma 1. *A pth-order product formula approximating evolution under a Hamiltonian $H = \sum_{\gamma=1}^{\Gamma} H_\gamma$, where all H_γ are geometrically local on a lattice of n qubits, has additive and multiplicative error with the following asymptotic scaling:*

$$\|\mathcal{A}(t)\|, \|\mathcal{M}(t)\| = O(nt^{p+1}). \tag{16}$$

Proof. By the results in [1] we have the bound

$$\|\mathcal{A}(t)\|, \|\mathcal{M}(t)\| = O\left(\sum_{\gamma_1,\ldots,\gamma_{p+1}=1}^{\Gamma} \|W_{\gamma_1,\ldots,\gamma_{p+1}}\| t^{p+1}\right), \tag{17}$$

where $W_{\gamma_1,\ldots,\gamma_{p+1}} = [H_{\gamma_{p+1}}, \ldots [H_{\gamma_2}, H_{\gamma_1}] \ldots]$. As any given H_γ is geometrically local it can be written as in Supplementary Equation (14) as

$$H = \sum_{Z \subset \Lambda^d} H_{\gamma,Z}, \tag{18}$$

where the $H_{\gamma,Z}$ act on subsets Z of maximum diameter R. For lattice site i let us define

$$H_\gamma^i := \sum_{Z \ni i} \frac{1}{|Z|} H_{\gamma,Z}, \tag{19}$$

i.e., the sum of all local terms in H_γ that act on site i, each divided by the size of their support set; this accounts for multi-counting and means that we can write

$$H_\gamma = \sum_{i \in \Lambda^d} H_\gamma^i. \tag{20}$$

For each H_γ^i we have $\|H_\gamma^i\| \leq C$ for some constant C dependent on R and d. We may now write

$$W_{\gamma_1,\ldots,\gamma_{p+1}} = \sum_{i_1,\ldots,i_{p+1} \in \Lambda^d} [H_{\gamma_{p+1}}^{i_{p+1}}, \ldots [H_{\gamma_2}^{i_2}, H_{\gamma_1}^{i_1}] \ldots]. \tag{21}$$

We can simplify this expression by omitting terms that are zero due to lack of shared support. The commutator $[H_{\gamma_2}^{i_2}, H_{\gamma_1}^{i_1}]$ vanishes if i_2 is more than $2R$ away from i_1 as no part of the two arguments will overlap. Furthermore, assuming the inside commutator is nonzero, $[H_{\gamma_3}^{i_3}, [H_{\gamma_2}^{i_2}, H_{\gamma_1}^{i_1}]]$ vanishes if i_3 is more than $3R$ away from i_1, because at that distance, $H_{\gamma_3}^{i_3}$ only overlaps with the parts of $H_{\gamma_2}^{i_2}$ that do not overlap with $H_{\gamma_1}^{i_1}$. By similar logic, i_4 must be within $4R$ of i_1, and so on. We can then reduce the sum to

$$W_{\gamma_1,\ldots,\gamma_{p+1}} = \sum_{i_{p+1}: D(i_{p+1}, i_1) \leq (p+1)R} \cdots \sum_{i_2: D(i_2,i_1) \leq 2R} \sum_{i_1 \in \Lambda^d} [H_{\gamma_{p+1}}^{i_{p+1}}, \ldots, [H_{\gamma_2}^{i_2}, H_{\gamma_1}^{i_1}] \ldots]. \tag{22}$$

The number of lattice points within a fixed distance of a given point is constant, so the sum over $\|W_{\gamma_1,\ldots,\gamma_{p+1}}\|$ in Supplementary Equation (17) simply reduces to a sum of constants over the points $i_1 \in \Lambda^d$, which is proportional to the number of lattice points n. The result follows. □

Supplementary Corollary 2. *For a pth-order product formula for a Hamiltonian H as described above, to simulate evolution for time t with accuracy ϵ, it suffices to use r iterations of the product formula, where*

$$r = O\left(\frac{n^{1/p}}{\epsilon^{1/p}} t^{1+1/p}\right). \tag{23}$$

The above results apply equally to THRIFT product formulas, using either the error scaling given in Supplementary Equation (6) or in Supplementary Equation (11). Furthermore, linear scaling of the spectral error with system size also applies to the average-case error by a similar argument.

3 Limits on error scaling in α for time-dependent product formulas

In this section we establish limitations on how well time-dependent product formulas can approximate Hamiltonian dynamics as a function of α, a scaling factor for the Hamiltonian. Such an evolution is obtained in THRIFT when approximating the time-dependent part of an interaction-picture evolution operator via a time-dependent product formula. In particular, the Hamiltonian in this time-dependent part is scaled by α, so these results provide limitations on the α-dependence of THRIFT.

Supplementary Theorem 3. *For a Hamiltonian of the form*

$$H(t) = \sum_{\gamma=1}^{\Gamma} \alpha H_\gamma(t), \tag{24}$$

consider a time-dependent product formula of the form

$$S(t) = \prod_{v=1}^{\Upsilon} \prod_{\gamma=1}^{\Gamma} \mathcal{T} e^{\int_{a_{v-1}t}^{a_v t} \alpha H_{\pi_v(\gamma)}(s) ds}, \tag{25}$$

where π_v are permutations of the indices γ and a_v are real numbers defining time intervals $[a_{v-1}t, a_v t]$. There is no such product formula for which

$$\left\| S(t) - \mathcal{T} e^{\int_0^t \alpha H(s) ds} \right\| = O(\alpha^k) \tag{26}$$

for $k > 2$ and $t \neq 0$.

Proof. We expand the terms of Supplementary Equation (26) into integral series, yielding Taylor series in α that can be compared term-by-term. Up to second order, the Dyson series for evolution under $H(t)$ over the interval $[0, t]$ is

$$\mathcal{T} e^{\int_0^t \alpha H(s) ds} = 1 + \alpha \int_0^t ds_1 \sum_\gamma H_\gamma(s_1) + \alpha^2 \int_0^t ds_1 \int_0^{s_1} ds_2 \sum_{\gamma_1, \gamma_2} H_{\gamma_1}(s_1) H_{\gamma_2}(s_2) + O(\alpha^3). \tag{27}$$

Expanding each time-ordered integral in $S(t)$ and collecting powers of α gives the second-order expansion

$$\begin{aligned} S(t) = 1 &+ \alpha \sum_v \int_{a_{v-1}t}^{a_v t} ds_1 \sum_\gamma H_\gamma(s_1) \\ &+ \alpha^2 \sum_v \int_{a_{v-1}t}^{a_v t} ds_1 \int_{a_{v-1}t}^{s_1} ds_2 \sum_\gamma H_\gamma(s_1) H_\gamma(s_2) \\ &+ \alpha^2 \sum_v \int_{a_{v-1}t}^{a_v t} ds_1 \int_{a_{v-1}t}^{a_v t} ds_2 \sum_{\gamma_1 < \gamma_2} H_{\pi_v(\gamma_1)}(s_1) H_{\pi_v(\gamma_2)}(s_2) \\ &+ \alpha^2 \sum_{v > u} \int_{a_{v-1}t}^{a_v t} ds_1 \int_{a_{u-1}t}^{a_u t} ds_2 \sum_{\gamma_1, \gamma_2} H_{\gamma_1}(s_1) H_{\gamma_2}(s_2) + O(\alpha^3). \end{aligned} \tag{28}$$

Combining integrals with matching boundaries and using the fact that $v > u$, we may rewrite this as

$$\begin{aligned} S(t) = 1 &+ \alpha \int_{a_0 t}^{a_\Upsilon t} ds_1 \sum_\gamma H_\gamma(s_1) \\ &+ \alpha^2 \int_{a_0 t}^{a_\Upsilon t} ds_1 \int_{a_0 t}^{s_1} ds_2 \sum_\gamma H_\gamma(s_1) H_\gamma(s_2) \\ &+ \alpha^2 \sum_v \int_{a_{v-1}t}^{a_v t} ds_1 \int_{a_{v-1}t}^{a_v t} ds_2 \sum_{\gamma_1 < \gamma_2} H_{\pi_v(\gamma_1)}(s_1) H_{\pi_v(\gamma_2)}(s_2) \\ &+ \alpha^2 \sum_v \int_{a_0 t}^{a_v t} ds_1 \int_{a_0 t}^{a_{v-1} t} ds_2 \sum_{\gamma_1 \neq \gamma_2} H_{\gamma_1}(s_1) H_{\gamma_2}(s_2) + O(\alpha^3). \end{aligned} \tag{29}$$

Clearly, for $S(t)$ to approximate evolution under $H(t)$ over $[0, t]$ to first order in α, we must have $a_0 = 0$ and $a_\Upsilon = 1$.

Then we find the following expression for the difference between $S(t)$ and the ideal evolution:

$$
\begin{aligned}
S(t) - \mathcal{T}e^{\int_0^t \alpha H(s)ds} = \alpha^2 \sum_v \int_{a_{v-1}t}^{a_v t} ds_1 \int_{a_{v-1}t}^{a_v t} ds_2 \sum_{\gamma_1<\gamma_2} H_{\pi_v(\gamma_1)}(s_1) H_{\pi_v(\gamma_2)}(s_2) \\
+ \alpha^2 \sum_v \int_0^{a_v t} ds_1 \int_0^{a_{v-1}t} ds_2 \sum_{\gamma_1\neq\gamma_2} H_{\gamma_1}(s_1) H_{\gamma_2}(s_2) \\
- \alpha^2 \int_0^t ds_1 \int_0^{s_1} ds_2 \sum_{\gamma_1\neq\gamma_2} H_{\gamma_1}(s_1) H_{\gamma_2}(s_2) + O(\alpha^3).
\end{aligned}
\tag{30}
$$

By inserting

$$
0 = \alpha^2 \sum_v \int_{a_{v-1}t}^{a_v t} ds_1 \int_{a_{v-1}t}^{s_1} ds_2 \sum_{\gamma_1<\gamma_2} \left(H_{\pi_v(\gamma_2)}(s_1) H_{\pi_v(\gamma_1)}(s_2) - H_{\pi_v(\gamma_2)}(s_1) H_{\pi_v(\gamma_1)}, (s_2) \right), \tag{31}
$$

doing some algebra, and relabelling integral variables where needed, we finally arrive at a more compact form for the error at second order, namely

$$
\begin{aligned}
S(t) - \mathcal{T}e^{\int_0^t \alpha H(s)ds} = \Delta\alpha^2 + O(\alpha^3) \\
\text{with} \quad \Delta = \sum_v \int_{a_{v-1}t}^{a_v t} ds_1 \int_{s_1}^{a_v t} ds_2 \sum_{\gamma_1<\gamma_2} [H_{\pi_v(\gamma_1)}(s_1), H_{\pi_v(\gamma_2)}(s_2)].
\end{aligned}
\tag{32}
$$

The question then becomes: is there a generic set of parameters $\{a_v\}$ and $\{\pi_v\}$ such that Δ vanishes?[1] Relabelling variables and using the antisymmetry of the commutator, we may write

$$
\Delta = \sum_v \sum_{\gamma_1<\gamma_2} \mathcal{S}(\pi_v, \gamma_1, \gamma_2) \int_{a_{v-1}t}^{a_v t} ds_{\Pi^v_{\gamma_1,\gamma_2}(1)} \int_{s_{\Pi^v_{\gamma_1,\gamma_2}(1)}}^{a_v t} ds_{\Pi^v_{\gamma_1,\gamma_2}(2)} [H_{\gamma_1}(s_1), H_{\gamma_2}(s_2)] \tag{33}
$$

where the function $\mathcal{S}(\pi_v, \gamma_1, \gamma_2)$ is -1 if the permutation π_v switches the order of γ_1, γ_2 and $+1$ otherwise, and $\Pi^v_{\gamma_1,\gamma_2}$ is a permutation of the indices 1 and 2 defined as

$$
\Pi^v_{\gamma_1,\gamma_2}((1,2)) = \begin{cases} (1,2) & \text{if } \pi_v((\gamma_1,\gamma_2)) = (\gamma_1,\gamma_2), \\ (2,1) & \text{if } \pi_v((\gamma_1,\gamma_2)) = (\gamma_2,\gamma_1). \end{cases} \tag{34}
$$

We may further rewrite this as

$$
\Delta = \sum_{\gamma_1<\gamma_2} \iint_{[t_{\min},t_{\max}]^2} ds_1 ds_2 \sum_v f^v_{\gamma_1,\gamma_2}(s_1,s_2) [H_{\gamma_1}(s_1), H_{\gamma_2}(s_2)], \tag{35}
$$

where $t_{\min}$ and $t_{\max}$ are the minimum and maximum values of $\{a_v t\}_{v=1}^{\Upsilon}$ and $f^v_{\gamma_1,\gamma_2}$ are functions that are zero everywhere except for the corresponding region of integration in Supplementary Equation (33) where they take the value of $\mathcal{S}(\pi_v, \gamma_1, \gamma_2)$. The possible forms of these functions are visualised in Supplementary Table 1. For Δ to vanish, each term in the sum over $\gamma_1 < \gamma_2$ must vanish. Furthermore, for arbitrary time-dependent Hamiltonians, this requires that the respective sums $\sum_v f^v_{\gamma_1,\gamma_2}(s_1,s_2)$ vanish for all s_1, s_2. By inspection of the forms of these functions in Supplementary Table 1, it is clear that this requires every time step $(a_{v-1}t, a_v t)$ to have a step of the same size in the reverse direction, meaning that Δ only vanishes for evolution over zero time. Supplementary Theorem 3 then follows. □

In fact, Supplementary Theorem 3 holds even with the further restriction that the time-dependence of the Hamiltonian arises via conjugation by some fixed Hamiltonian dynamics, as in the case of THRIFT.

[1] Technically, for the error to agree up to second order, Δ only needs to be $O(\alpha)$, however this α dependence would be contained in the choice of parameters which can be chosen independently of the value of α in the Hamiltonian. Accordingly, they can then be treated as depending on a separate parameter β whose value can be fixed to give the best upper bound for Δ, hence the treatment of $\{a_v\}$ and $\{\pi_v\}$ as constants.

category	function definition	non-zero region
$\Pi^v_{\gamma_1,\gamma_2}$ even $a_{v-1}t < a_v t$	$f^v_{\gamma_1,\gamma_2}(s_1,s_2) = \begin{cases} 1 & : \quad a_{v-1}t < s_1 < s_2 < a_v t \\ 0 & : \quad \text{otherwise} \end{cases}$	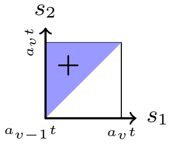
$\Pi^v_{\gamma_1,\gamma_2}$ even $a_v t < a_{v-1}t$	$f^v_{\gamma_1,\gamma_2}(s_1,s_2) = \begin{cases} 1 & : \quad a_v t < s_2 < s_1 < a_{v-1}t \\ 0 & : \quad \text{otherwise} \end{cases}$	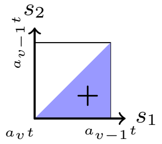
$\Pi^v_{\gamma_1,\gamma_2}$ odd $a_{v-1}t < a_v t$	$f^v_{\gamma_1,\gamma_2}(s_1,s_2) = \begin{cases} -1 & : \quad a_{v-1}t < s_2 < s_1 < a_v t \\ 0 & : \quad \text{otherwise} \end{cases}$	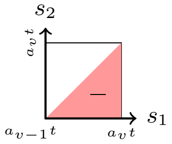
$\Pi^v_{\gamma_1,\gamma_2}$ odd $a_v t < a_{v-1}t$	$f^v_{\gamma_1,\gamma_2}(s_1,s_2) = \begin{cases} -1 & : \quad a_v t < s_1 < s_2 < a_{v-1}t \\ 0 & : \quad \text{otherwise} \end{cases}$	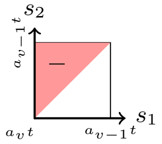

Supplementary Table 1: The form of $f^v_{\gamma_1,\gamma_2}$ depends on the parity of $\Pi^v_{\gamma_1,\gamma_2}$, i.e., whether it switches 1 and 2, and whether $a_{v-1}t < a_v t$. All four possibilities are shown, along with a visualisation of their non-zero region as a shaded area on the s_1-s_2 plane. Blue shading with a "+" indicates a value of $+1$ and, likewise, red with a "−" a value of -1.

Supplementary Theorem 4. *Supplementary Theorem 3 also holds for the restricted case where $\tilde{H}_\gamma(t) = e^{H_0 t} H_\gamma(0) e^{-H_0 t}$ for some fixed H_0.*

We show this by adapting the above proof of Supplementary Theorem 3. The sum over $\gamma_1 < \gamma_2$ must still vanish term-wise, so let us simplify by analysing the $\gamma_1 = 1$, $\gamma_2 = 2$ term, denoting it $T(H_1, H_2)$. Let us simplify further by writing the sum over $f^v_{1,2}$ as a single function F and relabelling $s_1 \to x$, $s_2 \to y$, so we have

$$T(H_1, H_2) = \iint_{S^2} dx dy F(x,y)[\tilde{H}_1(x), \tilde{H}_2(y)], \tag{36}$$

where $S := [t_{\min}, t_{\max}]$. We have

$$\tilde{H}_1(t) = e^{H_0 t} H_1(0) e^{-H_0 t}, \quad \tilde{H}_2(t) = e^{H_0 t} H_2(0) e^{-H_0 t}. \tag{37}$$

Supplementary Lemma 5. *If $T(H_1, H_2) = 0$ for any H_1 with $[H_0, \tilde{H}_1(0)] \neq 0$, then $T(H_1', H_2) = 0$ for any H_1' with $[H_0, \tilde{H}_1'(0)] = 0$.*

Proof. For H_1' such that $[H_0, \tilde{H}_1'(0)] = 0$ define $\tilde{H}_1''(x) = \tilde{H}_1(x) + A(x)$ where $A(x)$ is some operator-valued function with $[H_0, A(0)] \neq 0$. Then by linearity we have

$$T(H_1', H_2) = T(H_1'', H_2) + T(A, H_2) = 0. \tag{38}$$

As $T(A, H_2) = 0$ by hypothesis, we have $T(H_1', H_2) = 0$. ◻

Supplementary Lemma 6. *If $T(H_1, H_2) = 0$ for any H_1, H_2, then for all $k \in \mathbb{N}$,*

$$\iint_{S^2} dx dy F(x,y) x^k = \iint_{S^2} dx dy F(x,y) y^k = 0. \tag{39}$$

Proof. Let $[\tilde{H}_1(0), H_0] = 0$ and $H_0 = \lambda H_0'$. Expand Δ as a power series in λ to get

$$\Delta = \sum_k^{\infty} \frac{1}{k!} \iint_{S^2} dxdyF(x,y)[H_1, \mathrm{ad}_{H_0'}^k H_2] y^k \lambda^k = 0 \tag{40}$$

where $\mathrm{ad}_A B = [A,B]$ and we write $\tilde{H}_1(0)$ as H_1, likewise for H_2. This series must vanish term-by-term with λ, so we have

$$[H_1, \mathrm{ad}_{H_0'}^k H_2] \iint_{S^2} dxdyF(x,y)y^k = 0 \quad \forall k \in \mathbb{N}. \tag{41}$$

Let H_0', H_1, H_2 be Paulis that pairwise anticommute except H_0 and H_1. Then $\mathrm{ad}_{H_0'}^k H_2 = 2^k H_0'^k H_2$, meaning $[H_1, \mathrm{ad}_{H'}^k H_2] \neq 0$ for all $k \in \mathbb{N}$. For $T(H_1, H_2) = 0$ to hold in general, the above integral must then vanish for all k. The same argument applies for x. □

Supplementary Lemma 7. *If for all $k \in \mathbb{N}$,*

$$\int_{[a,b]} dxf(x)x^k = 0, \tag{42}$$

then

$$f(x) = 0 \quad \textit{almost everywhere in } [a,b], \tag{43}$$

i.e., it is nonzero on only a measure-zero subset of $[a,b]$.

Proof. The following proof is reproduced from [3] in more detail.

Let $f(x)$ be integrable over the interval $[a,b]$ and have the property that $\int_{[a,b]} dxf(x)x^k = 0$ for all $k \in \mathbb{N}$. It follows that $\int_{[a,b]} dxf(x)p(x) = 0$ for any polynomial p. The polynomials are dense in the set of continuous functions on $[a,b]$, so it also follows that for any continuous function g, $\int_{[a,b]} dxf(x)g(x) = 0$.

Assume now that f is not zero almost everywhere. Then the set $\{x : f(x) > 0\}$ has finite measure. One can then find δ such that $\{x : f(x) > \delta\}$ has finite measure. By the regularity of the Lebesgue measure, one can choose a compact set K and open set V such that $K \subset \{x : f(x) > \delta\} \subset V$ and the measure of $V \setminus K$ is arbitrarily small. Let g be a continuous function such that $0 \leq g(x) \leq 1$ which is equal to 1 on K and 0 outside of V. Then we have

$$\left| \int_{[a,b]} dxg(x)f(x) \right| = \left| \int_V dxg(x)f(x) \right| = \left| \int_K dxg(x)f(x) + \int_{V\setminus K} dxg(x)f(x) \right|. \tag{44}$$

By the (reverse) triangle inequality, we have

$$\begin{aligned} \left| \int_{[a,b]} dxg(x)f(x) \right| &\geq \left| \int_K dxg(x)f(x) \right| - \left| \int_{V\setminus K} dxg(x)f(x) \right| \\ &\geq \left| \int_K dxg(x)f(x) \right| - \int_{V\setminus K} dx|g(x)f(x)|. \end{aligned} \tag{45}$$

Now as g is 1 on K and $g < 1$ for some region on V, we have

$$\left| \int_{[a,b]} dxg(x)f(x) \right| \geq \left| \int_K dxf(x) \right| - \int_{V\setminus K} dx|f(x)|. \tag{46}$$

As noted before, $f > \delta$ on K, so

$$\left| \int_{[a,b]} dxg(x)f(x) \right| \geq \delta m(K) - \int_{V\setminus K} dx|f(x)| \tag{47}$$

where $m(K) > 0$ is the measure of K. Since $V \setminus K$ can be made arbitrarily small, we can take the right-hand side of the inequality to be positive, meaning that $\left| \int_{[a,b]} dxg(x)f(x) \right|$ for some continuous g, which is a contradiction. □

Supplementary Corollary 8. *If for all $k \in \mathbb{N}$,*

$$\iint_{S^2} dxdyF(x,y)x^k = 0, \tag{48}$$

then as a function of x,

$$\int_S dyF(x,y) = 0 \quad \textit{almost everywhere in } S. \tag{49}$$

The same holds when y and x are exchanged.

Proof. Set

$$f(x) = \int_S dyF(x,y) \tag{50}$$

and $[a,b] = S$. The result follows immediately from Supplementary Lemma 7. □

Supplementary Lemma 9. *If $T(H_1, H_2) = 0$ for all $H_0, \tilde{H}_1(x), \tilde{H}_2(y)$ as defined above, then $a_\Upsilon t = a_0 t$.*

Proof. Assume $a_\Upsilon t \neq a_0 t$. The intervals $[a_{v-1}t, a_v t]$ are "steps" in a path from $a_0 t$ to $a_\Upsilon t$, so any point in $S \setminus \{a_v t\}$ is contained within an odd number of these intervals as the path must cross it an odd number of times. Consider the open interval $(a_v t, a'_v t)$ where $a'_v = \min\{a_u : a_u > a_v\}$. Any point in this set is contained in the same set of "step" intervals. There must exist a v for which $(a_v t, a'_v t) \cap [a_0 t, a_\Upsilon t] \neq \varnothing$, so there exists a finite-measure set of points that are all contained within the same set of steps M where $|M|$ is odd.

As $F(x,y)$ is a sum of the functions in Supplementary Table 1, we can see that for a point x' in this set, the integral $\int_S dyF(x',y)$ takes the form

$$\int_S dyF(x',y) = \sum_{v:[a_{v-1}t, a_v t] \in M} s_v(x' - T_v) \tag{51}$$

where $s_v \in \{\pm 1\}$ and $T_v \in \{a_{v-1}t, a_v t\}$. Consider now the integral for $x' + c \in (a_v t, a'_v t)$,

$$\begin{aligned} \int_S dyF(x'+c, y) &= \sum_{v:[a_{v-1}t, a_v t] \in M} s_v(x' + c - T_v) \\ &= \sum_{v:[a_{v-1}t, a_v t] \in M} s_v(x' - T_v) + \sum_{v:[a_{v-1}t, a_v t] \in M} s_v c. \end{aligned} \tag{52}$$

As $|M|$ is odd and s_v are signs,

$$\left| \sum_{v:[a_{v-1}t, a_v t] \in M} s_v c \right| \geq 1, \tag{53}$$

and $\int_S dyF(x,y)$ is non-zero on a finite measure set, contradicting Supplementary Corollary 8. □

We are now ready to prove Supplementary Theorem 4.

Proof of Supplementary Theorem 4. Recall that for the product formula in Supplementary Equation (25) to agree up to α^2 for arbitrary times, the quantity

$$\Delta = \sum_{\gamma_1 < \gamma_2} \iint_{S^2} ds_1 ds_2 F(s_1, s_2)[H_{\gamma_1}, H_{\gamma_2}] \tag{54}$$

must vanish. This sum must vanish term-wise, so it suffices to consider the $\gamma_1 = 1$, $\gamma_2 = 2$ case. By Supplementary Lemma 9, this term may only vanish if $a_0 t = a_\Upsilon t$. As argued in the proof of Supplementary Theorem 3, first-order agreement requires $a_0 = 0$ and $a_\Upsilon = 1$, so second-order agreement can only hold if $t = 0$. □

Supplementary Note 2 – Convergence of Magnus expansion

A simple proof of the convergence of the Magnus expansion is given in [4]. Here we reproduce it for completeness. The first ingredient is the following lemma.

Supplementary Lemma 10 (A Bihari-type inequality [4])**.** *Let $h, v \in C(0,T)$ (where $C(0,T)$ denotes functions with a continuous first derivative on the interval $[0,T]$) be integrable positive functions and let $g \in C(0,T)$ be a non-decreasing positive function. Then*

$$h(x) \leq \int_0^x v(s)g(h(s))ds \tag{55}$$

for $x \in [0,T]$ implies that $h(x) \leq \int_0^x v(s)g(h(s))ds \leq G^{-1}(\int_0^x v(s)ds)$, where G^{-1} is the inverse function of $G(s) = \int_0^s \frac{ds}{g(s)}$.

Proof. Define $f(x) := \int_0^x v(x)g(h(x))dx$, so $\frac{df}{dx} = v(x)g(h(x))$. Using Supplementary Equation (55), $h \leq f$, which implies $g(h(x)) \leq g(f(x))$ as g is non-decreasing. Therefore $\frac{df}{dx} \leq v(x)g(f(x))$. Dividing by g and integrating by substitution, we have

$$\int_0^{f(t)} \frac{ds}{g(s)} \leq \int_0^t v(x)dx \quad \Rightarrow \quad G(f(t)) \leq \int_0^t v(x)dx. \tag{56}$$

Applying the inverse of G and using $h \leq f$ completes the proof. □

Supplementary Theorem 11. *The Magnus expansion $\Omega(t)$, defined by $\mathcal{T}e^{-i\alpha \int_0^t \tilde{H}_1(s)ds} = e^{\Omega(t)}$ and the series in Eq. (32) in the main text, converges for $|\alpha| \int_0^t \|\tilde{H}_1(s)\|ds \leq 1.08687\ldots$.*

Proof. Starting from the definition of the Magnus operator Eq. (32) in the main text, the triangle inequality gives

$$\|\Omega(t)\| \leq |\alpha| \int_0^t \sum_{k=0}^{\infty} \frac{|b_k|}{k!}(2\|\Omega(s)\|)^k \|\tilde{H}_1(s)\|ds = |\alpha| \int_0^t g(2\|\Omega(s)\|)\|\tilde{H}_1(s)\|ds. \tag{57}$$

As g is a nondecreasing positive function in the interval $[0, 2\pi)$, we can apply Supplementary Lemma 10, giving

$$\|\Omega(t)\| \leq \frac{1}{2}G^{-1}\left(2|\alpha| \int_0^t \|\tilde{H}_1(s)\|ds\right). \tag{58}$$

This implies that $\|\Omega(t)\|$ is bounded as long as

$$|\alpha| \int_0^t \|\tilde{H}_1(s)\|ds \leq \frac{1}{2}G(2\pi) = \frac{1}{2}\int_0^{2\pi} \frac{ds}{2 + \frac{x}{2}(1 - \cot(x/2))} = 1.08687\ldots, \tag{59}$$

as claimed. □

The proof of the Magnus-THRIFT approximation theorem (Theorem 3 in the main text) uses Lemma 4 in the main text, which we now prove.

Supplementary Lemma 12. *For $1 \leq l$, $\|\tilde{\Omega}_l(t)\| \leq \frac{1}{2}x_l(2\int_0^t \|\tilde{H}_1(s)\|ds)^l$, where x_l is the coefficient of s^l in the expansion of $G^{-1}(s) = \sum_{m=1}^{\infty} x_m s^m$, the inverse function of $G(s) = \int_0^s (2 + \frac{x}{2}(1 - \cot(x/2))^{-1}dx$.*

Proof. We proceed by induction. First, as $\tilde{\Omega}_1(t) = -i\int_0^t \tilde{H}_1(s)ds$, we have

$$\|\tilde{\Omega}_1(t)\| \leq \int_0^t \|\tilde{H}_1(s)\|ds = \frac{x_1}{2}\left(2\int_0^t \|\tilde{H}_1(s)\|ds\right) \tag{60}$$

with $x_1 = 1$. The induction hypothesis is $\|\tilde{\Omega}_l(t)\| \leq \frac{1}{2}x_l(2\int_0^t \|\tilde{H}_1(s)\|ds)^l$ for $1 \leq l \leq n$. To prove the induction step, we integrate Eq. (33) in the main text and use the triangle inequality, leading to

$$\begin{aligned}\|\tilde{\Omega}_{n+1}(t)\| &\leq \sum_{j=1}^{n} \frac{|b_j|}{j!} \sum_{\substack{k_1+k_2+\cdots+k_j=n \\ k_1,k_2,\ldots,k_j\geq 1}} \int_0^t 2^j \prod_{m=1}^{j} \|\tilde{\Omega}_{k_m}(s)\| \|\tilde{H}_1(s)\| ds, \\ &= \sum_{j=1}^{n} \frac{|b_j|}{j!} \int_0^t 2^j \hat{B}_{n,j}(\|\tilde{\Omega}_1(s)\|, \ldots, \|\tilde{\Omega}_{n-j+1}(s)\|) \|\tilde{H}_1(s)\| ds,\end{aligned} \tag{61}$$

where we have introduced the ordinary Bell polynomials [5, 6], defined by

$$\hat{B}_{n,j}(x_1, x_2, \ldots, x_{n-j+1}) := \frac{1}{n!} \frac{\partial^n}{\partial \alpha^n} \left(\sum_{k=1}^{\infty} \alpha^k x_k \right)^j \Bigg|_{\alpha=0} = \sum_{\substack{k_1+k_2+\cdots+k_j=n \\ k_1,k_2,\ldots,k_j\geq 1}} \prod_{m=1}^{j} x_{k_m}. \tag{62}$$

Using the induction hypothesis on Supplementary Equation (61) and that $\hat{B}_{n,j}(rx_1, r^2x_2 \ldots, r^{n-k+1}x_{n-k+1}) = r^n \hat{B}_{n,j}(x_1, x_2 \ldots, x_{n-k+1})$, which follows from the definition Supplementary Equation (62), we have

$$\|\tilde{\Omega}_{n+1}(t)\| \leq \left(\int_0^t \left(2 \int_0^s \|\tilde{H}_1(y)\| dy \right)^n \|\tilde{H}_1(s)\| ds \right) \sum_{j=1}^{n} \frac{|b_j|}{j!} \hat{B}(x_1, \ldots, x_{n-j+1}), \tag{63}$$

$$= \left(\frac{1}{2} \int_0^t \frac{d}{ds} \frac{(2\int_0^s \|\tilde{H}_1(x)\| dx)^{n+1}}{n+1} ds \right) \sum_{j=1}^{n} \frac{|b_j|}{j!} \hat{B}_{n,j}(x_1, \ldots, x_{n-j+1}) \quad \text{using the chain rule,} \tag{64}$$

$$= \frac{(2\int_0^t \|\tilde{H}_1(x)\| dx)^{n+1}}{n+1} \frac{1}{2} \sum_{j=1}^{n} \frac{|b_j|}{j!} \hat{B}_{n,j}(x_1, \ldots, x_{n-j+1}) \quad \text{using the fundamental theorem of calculus.} \tag{65}$$

To finish the proof, we show that the factor $\frac{1}{(n+1)} \sum_{j=1}^{n} \frac{|b_j|}{j!} \hat{B}_{n,j}(x_1, \ldots, x_{n-j+1})$ corresponds to the coefficient of z^{n+1} in the series expansion $G^{-1}(z) = \sum_{m=1}^{\infty} z^m x_m$, given that $\{x_j\}_{j=1}^{n}$ are also coefficients of G^{-1}. That can be shown as follows:

$$X_{n+1} := \frac{1}{n+1} \sum_{j=1}^{n} \frac{|b_j|}{j!} \hat{B}_{n,j}(x_1, \ldots, x_{n-j+1}) = \frac{1}{(n+1)!} \sum_{j=1}^{n} |b_j| B_{n,j}(1!x_1, 2!x_2, \ldots, (n-j+1)!x_{n-k+1}) \tag{66}$$

where we used the relation

$$\frac{n!}{j!} \hat{B}_{n,j}(x_1, \ldots, x_{n-j+1}) = B_{n,j}(1!x_1, 2!x_2, \ldots, (n-j+1)!x_{n-k+1}) \tag{67}$$

between the ordinary Bell polynomials $\hat{B}_{n,k}$ and the exponential Bell polynomials $B_{n,k}$ [6]. Now note that $G^{-1}(z) = \sum_{n=1}^{\infty} z^n x_n$ implies $\frac{d^n G^{-1}(0)}{dz^n} = n! x_n$, so we can write Supplementary Equation (66) as

$$\begin{aligned}X_{n+1} &= \frac{1}{(n+1)!} \sum_{j=1}^{n} |b_j| B_{n,j} \left(\frac{dG^{-1}(0)}{dz}, \frac{d^2G^{-1}(0)}{dz^2}, \ldots, \frac{d^{n-j+1}G^{-1}(0)}{dz^{n-j+1}} \right), \\ &= \frac{1}{(n+1)!} \sum_{j=1}^{n} \frac{d^j g(0)}{dz^j} B_{n,j} \left(\frac{dG^{-1}(0)}{dz}, \frac{d^2G^{-1}(0)}{dz^2}, \ldots, \frac{d^{n-j+1}G^{-1}(0)}{dz^{n-j+1}} \right),\end{aligned} \tag{68}$$

with $g(z) = \sum_{j=0}^{\infty} \frac{|b_j|}{j!} z^j = 2 + \frac{z}{2}(1 - \cot(z/2))$. Finally, using the derivative rule for inverse functions $\frac{dG^{-1}(z)}{dz} = \frac{1}{G'(G^{-1}(z))}$ and the definition of $G(z) = \int_0^z (g(s))^{-1} ds$, we have $\frac{dG^{-1}(z)}{dz} = g(G^{-1}(z))$. In general,

$$\frac{d^{n+1}}{dz^{n+1}}(G^{-1}(z)) = \frac{d^n}{dz^n}(g(G^{-1}(z))) = \sum_{k=1}^{n} \frac{d^j}{dz^j}(g(G^{-1}(z)) B_{n,k} \left(\frac{dG^{-1}(z)}{dz}, \frac{d^2G^{-1}(z)}{dz^2}, \ldots, \frac{d^{n-k+1}G^{-1}(z)}{dz^{n-k+1}} \right) \tag{69}$$

where we have used Faà di Bruno's identity for the generalised chain rule [7]. Comparing Supplementary Equation (68) and Supplementary Equation (69), we find

$$X_{n+1} = \frac{1}{(n+1)!}\frac{d^{n+1}}{dz^{n+1}}(G^{-1}(0)), \tag{70}$$

which is by definition x_{n+1}. Going back to Supplementary Equation (65), this implies

$$\|\tilde{\Omega}_{n+1}(t)\| \leq \frac{x_{n+1}}{2}\left(2\int_0^t \|\tilde{H}_1(x)\|dx\right)^{n+1} = \frac{1}{2(n+1)!}\frac{d^{n+1}}{dz^{n+1}}(G^{-1}(0))\left(2\int_0^t \|\tilde{H}_1(x)\|dx\right)^{n+1}. \tag{71}$$

This proves the induction step and hence the lemma. □

1 Fer-THRIFT

In this section we propose another algorithm which achieves $O(\alpha^{k+1}t^{k+1})$ error scaling. In particular, we note that we can bypass approximating the Magnus term $e^{\Omega^{[j]}(\alpha,t:\delta t)}$ in Eq. (14) in the main text by directly looking for an approximation of the time-ordered operator as a product of exponentials. This approach generates the following decomposition.

As in the Magnus-THRIFT case, the starting point is an approximation of the time-ordered operator in the interaction picture. For this approximation, Fer [8] postulated the form

$$\mathcal{T}e^{-i\int_0^t A(s)ds} = e^{-i\int_0^t A(s)ds}V(t). \tag{72}$$

This implies the equation

$$\frac{d}{dt}V = \left[-ie^{i\int_0^t A(s)ds}A(t)e^{-i\int_0^t A(s)ds} - e^{i\int_0^t A(s)ds}\frac{d}{dt}e^{-i\int_0^t A(s)ds}\right]V =: -iA_1(t)V, \tag{73}$$

which can be formally solved as $V = \mathcal{T}e^{-i\int_0^t A_1(s)ds}$. Repeating this procedure k times gives

$$\mathcal{T}e^{-i\int_0^t A(s)} = \prod_{j=0}^{k-1} e^{-i\int_0^t A_j(s)ds}V_k, \tag{74}$$

where $A_0 := A$ and

$$\begin{aligned} A_j(t) &= e^{i\int_0^t A_{j-1}(s)ds}A_{j-1}(t)e^{-i\int_0^t A_{j-1}(s)ds} - ie^{i\int_0^t A_{j-1}(s)ds}\frac{d}{dt}e^{-i\int_0^t A_{j-1}(s)}ds \\ &= \sum_{m=1}^{\infty}(-1)^m\frac{m}{(m+1)!}\mathrm{ad}^m_{-i\int_0^t A_{j-1}(s)ds}(A_{j-1}(t)). \end{aligned} \tag{75}$$

Setting $V_k = 1$ truncates this product, giving an approximation of order $O(t^{2^{k+1}-1})$ [9].

This analysis can be modified slightly to determine how the error depends on a scaling factor α by making the substitution $A_0 \to \alpha A_0$. For the following we absorb the factor of $-i$ into A_0 as it does not change the analysis.

Supplementary Lemma 13. *Let $A_0(t)$ be an operator-valued function that is analytic in t over the reals. For a real scaling factor α, define $\alpha A_k(t)$ recursively as*

$$\alpha A_{k+1}(t) = \sum_{m=1}^{\infty}(-1)^m\frac{m}{(m+1)!}\mathrm{ad}^m_{\int_0^t \alpha A_k(s)ds}[\alpha A_k(t)]. \tag{76}$$

If $\alpha A_k(t) = O(\alpha^q t^p)$ then $\alpha A_{k+1}(t) = O(\alpha^{2q}t^{2p+2})$.

Proof. The proof is largely similar to the proof of Lemma 2 of [9], differing in the fact that it also tracks the scaling variable α. For notational compactness, let $\alpha B_k(t) = \int_0^t \alpha A_k(s) ds$.

By Lemma 1 of [9], αA_k is analytic in t over the reals for all k. As $A_k(t)$ has no dependence on α, they are also analytic over all α. We may then write

$$\begin{aligned} \alpha A_k(t) &= \sum_{i=0}^{2p+1} \frac{1}{i!} \alpha A_k^{(i)}(0) t^i + \alpha^q t^{2p+2} E_A(\alpha, t), \\ \alpha B_k(t) &= \sum_{i=1}^{2p+1} \frac{1}{i!} \alpha A_k^{(i-1)}(0) t^i + \alpha^q t^{2p+2} E_B(\alpha, t), \end{aligned} \tag{77}$$

where the superscripts of A_k denote derivatives with respect to t.

By the bilinearity of the commutator, we have

$$[\alpha B_k(t), \alpha A_k(t)] = \sum_{i=1}^{\infty} \sum_{j=0}^{\infty} \frac{1}{i!j!} [\alpha A_k^{(i-1)}(0), \alpha A_k^{(j)}(0)] t^{i+j} + \alpha^{2q} t^{2p+2} E_1(\alpha, t) \tag{78}$$

where we have used the fact that $\alpha A_k^{(i)}(0) \in O(\alpha^q)$. Reordering the summation gives

$$[\alpha B_k(t), \alpha A_k(t)] = \sum_{i=1}^{\infty} \frac{1}{i!} \left[\sum_{j=0}^{j} \binom{i}{j} [\alpha A_k^{(i-j-1)}(0), \alpha A_k^{(j)}(0)] \right] t^i + \alpha^{2q} t^{2p+2} E_2(\alpha, t). \tag{79}$$

As $\alpha A_k(t) = O(t^p)$, $\alpha A_k^{(j)}(0) = 0$ for $0 \le j \le p-1$, so for $i \le 2p$,

$$\sum_{j=0}^{j} \binom{i}{j} [\alpha A_k^{(i-j-1)}(0), \alpha A_k^{(j)}(0)] = 0 \tag{80}$$

and

$$\sum_{j=0}^{2p+1} \binom{2p+1}{j} [\alpha A_k^{(2p-j)}(0), \alpha A_k^{(j)}(0)] = \binom{2p+1}{p} [\alpha A_k^{(p)}(0), \alpha A_k^{(p)}(0)] = 0. \tag{81}$$

Therefore $[\alpha B_k(t), \alpha A_k(t)] = O(\alpha^{2q} t^{2p+2})$. For the nested commutators we have

$$\mathrm{ad}^m_{\alpha B_k(t)}[\alpha A_k(t)] = O(\alpha^{mk} t^{m(p+1)}) \tag{82}$$

because $\alpha B_k(t) = O(\alpha^q t^{p+1})$, so $\alpha A_{k+1}(t) = O(\alpha^{2q} t^{2p+2})$ as claimed. □

Supplementary Theorem 14. *Let*

$$\begin{aligned} U(t) &= \mathcal{T} e^{\int_0^t \alpha A_0(s) ds}, \\ U_F(t) &= \prod_{j=0}^{k-1} e^{\int_0^t \alpha A_j(s) ds}, \end{aligned} \tag{83}$$

with A_j defined as in Supplementary Lemma 13. Then

$$\|U_F(t) - U(t)\| = O(\alpha^{2^k} t^{2^{k+1}-1}). \tag{84}$$

Proof. The proof of Theorem 3 of [9] shows that

$$U_F(t) - U(t) = -\int_0^t U(t-\tau) U_F(\tau) \alpha A_k(\tau). \tag{85}$$

The bound follows since $U(t), U_F(t) = O(1)$ and $\alpha A_k(t) = O(\alpha^{2^k} t^{2^{k+1}-2})$. □

For an approximation of the total evolution in the interaction picture, we have the following.

Supplementary Corollary 15 (Fer-THRIFT decomposition)**.** *Consider a Hamiltonian $H = H_0 + \alpha H_1$, and let $\tilde{H}_1(t) = e^{itH_0} H_1 e^{-itH_0}$. Define*

$$U_F(t) = e^{-itH_0} \prod_{j=0}^{k-1} e^{-i\int_0^t A_j(s)ds}, \tag{86}$$

where $A_j(t)$ is defined recursively from Supplementary Equation (75) with $A_0(t) := \alpha\tilde{H}_1(t)$. Then $U_F(t)$ approximates $U(t) = e^{-itH}$ up to $O(\alpha^{2^k} t^{2^{k+1}-1})$ for small times t.

Note that the surprising scaling of this approach with t and α is due to the assumption that the unitaries $e^{-i\int_0^t A_j(s)ds}$ can be implemented exactly. In any actual implementation, these unitaries have to approximated up to the target error, thus recovering in practice the same scaling as Magnus-THRIFT. This is exemplified in the following algorithm.

Fer-THRIFT Algorithm

To approximate the time evolution generated by the Hamiltonian $H = H_0 + \alpha H_1$ for time T with precision $O(N(T\alpha/N)^{p+1})$, we perform the following:

1. Write the evolution operator $U(T) = e^{-iT(H_0+\alpha H_1)}$ in the interaction picture, with H_0 as the dominant part, i.e.,
$$U(T) = e^{-iTH_0} \mathcal{T} e^{-i\int_0^T \alpha\tilde{H}_1(t)}. \tag{87}$$
2. Slice the time T into N intervals:
$$\mathcal{T} e^{-i\int_0^T \alpha\tilde{H}_1(t)} = \prod_{k=1}^{N} \mathcal{T} e^{-i\int_{(k-1)\frac{T}{N}}^{k\frac{T}{N}} \alpha\tilde{H}_1(t)}. \tag{88}$$
3. Approximate the time-ordered exponential of a slice using its Fer expansion up to order $O((\frac{T}{N}\alpha)^p)$:
$$\mathcal{T} e^{-i\int_t^{t+\delta t} \alpha\tilde{H}_1(t)} = \prod_{j=0}^{\log(p)} \exp\left(-i\int_t^{t+\delta t} A_j(s)ds\right) + O((\delta t\alpha)^{p+1}). \tag{89}$$
4. Approximate each exponential in the product using a pth-order formula:
$$\exp\left(-i\int_t^{t+\delta t} A_j(s)ds\right) = S_p^j(t,\delta t) + O((\delta t\alpha)^{p+1}). \tag{90}$$

This procedure leads to the decomposition

$$U(T) = e^{-iTH_0} \prod_{k=1}^{N} \prod_{j=0}^{\log(p)} S_p^j\left((k-1)\frac{T}{N}, \frac{T}{N}\right) + O\left(N\left(\frac{T\alpha}{N}\right)^{p+1}\right). \tag{91}$$

Note that for the error in the resulting simulation to have the stated scaling, the unitary e^{-iTH_0} must be implemented with error at most $O((T\alpha)^{p+1})$.

Supplementary Note 3 – Circuit details for numerical implementations

In this section we discuss the circuit depth for the both Trotter and THRIFT algorithms, using arbitrary 2-qubit gates, for the transverse-field Ising model (1D and 2D cases), 1D Heisenberg model, and 1D Fermi-Hubbard model. We consider a Hamiltonian of the form $H = H_0 + \alpha H_1$, where H_0 is a sum of single-qubit terms (unless otherwise specified); $H_1 = \sum_{j=1}^{K} h_j$, with each h_j containing terms acting on disjoint qubits; and $\alpha \ll 1$. Exponentials of the terms in H_1, e^{-ih_jt}, can therefore be implemented simultaneously with $\mathcal{N}_j$ arbitrary 2-qubit gates. For all the models we consider, we have $\mathcal{N}_j = \mathcal{N}$, independent of j.

1 General facts about product formulas

1.1 Trotter formulas

The first-order Trotter approximation (Trotter 1) for the time-evolution operator $U = e^{-iHt}$ is

$$\mathcal{S}_1(t) = P_1^K(t), \tag{92}$$

with

$$P_a^b(z) = \left(\prod_{j=a}^{b-1} e^{-ih_j z}\right) e^{-iH_0 z} e^{-ih_b z}. \tag{93}$$

Since $e^{-iH_0 t}$ only requires single-qubit gates, Supplementary Equation (92) can be implemented with $K\mathcal{N}$ layers of arbitrary 2-qubit gates.

The second-order Trotter approximation (Trotter 2) can be written as

$$\mathcal{S}_2(t) = P_1^K(t/2) P_K^1(t/2) = P_1^{K-1}(t/2) e^{-ih_K t} P_{K-1}^1(t/2), \tag{94}$$

and can be implemented with $(2K-1)\mathcal{N}$ layers of arbitrary 2-qubit gates. Note that if the number of Trotter layers is $N > 1$, one can merge the last exponential of the $(i-1)$st step with the first of the (i)th step, giving a total arbitrary 2-qubit gate depth of $[(2K-2)N+1]\mathcal{N}$.

The fourth-order Trotter approximation (Trotter 4) can be obtained from Supplementary Equation (94) as [10]

$$\mathcal{S}_4(t) = \mathcal{S}_2(s_2 t)^2 \mathcal{S}_2((1-4s_2)t) \mathcal{S}_2(s_2 t)^2, \qquad \text{with } s_2 := (4 - \sqrt[3]{4})^{-1}. \tag{95}$$

The final term of each $\mathcal{S}_2(z)$ can be merged with the first term of the following $\mathcal{S}_2(z)$, so Supplementary Equation (95) can be implemented with $[5(2K-2)+1]\mathcal{N} = (10K-9)\mathcal{N}$ layers of arbitrary 2-qubit gates. As in the Trotter 2 case, if the number of Trotter layers is $N > 1$, one can merge the last time-evolution operator of the $(i-1)$st step with the first of the (i)th step. This gives a total arbitrary 2-qubit gate depth of $[5(2K-2)N+1]\mathcal{N}$.

Finally, the optimised eighth-order Trotter approximation (optimised Trotter 8) is given by Eq. (15) in [11]

$$\mathcal{S}_8(t) = \left(\prod_{j=1}^{m} \mathcal{S}_2(\omega_{m-j+1} t)\right) \mathcal{S}_2(\omega_0 t) \left(\prod_{j=1}^{m} \mathcal{S}_2(\omega_j t)\right), \tag{96}$$

with $m = 7$. Similarly to the previous case, one obtains that Supplementary Equation (96) can be implemented with $[15(2K-2)+1]\mathcal{N} = (30K-29)\mathcal{N}$ layers of arbitrary 2-qubit gates. If the number of Trotter layers is $N > 1$, one can merge the last time-evolution operator of the $(i-1)$st step with the first of the (i)th step. This gives a total arbitrary 2-qubit gate depth of $[15(2K-2)N+1]\mathcal{N}$.

1.2 The "small A" formula of Omelyan et al.

In Ref. [12], Omelyan et al. derive an optimised fourth-order product formula for a Hamiltonian $H = H_0 + \alpha H_1$ with $\alpha \ll 1$. The error achieved by this formula scales as $O(\alpha^2 t^5) + O(\alpha t^7)$. This implies that there exists a regime for small time t in which this formula achieves a scaling in α similar to THRIFT.

For a Hamiltonian $H = H_0 + \alpha H_1$, Omelyan et al.'s optmised formula can be written as [12, 13]

$$U_O(t) = e^{-ia_1 H_0 t} e^{-ib_1 \alpha H_1 t} e^{-ia_2 H_0 t} e^{-ib_2 \alpha H_1 t} e^{-ia_3 H_0 t} e^{-ib_2 \alpha H_1 t} e^{-ia_2 H_0 t} e^{-ib_1 \alpha H_1 t} e^{-ia_1 H_0 t}, \tag{97}$$

where the numerically determined coefficients are

$$\begin{aligned} a_1 &= 0.5316386245813512, \\ b_1 &= -0.04375142191737413, \\ a_2 &= -0.3086019704406066, \\ b_2 &= \frac{1}{2} - b_1, \\ a_3 &= 1 - 2\sum_{i=1}^{2} a_i. \end{aligned} \tag{98}$$

Since, for all the systems considered in this work, we have $H = \sum_{k=1}^{\Lambda} h_k$ with $\Lambda \geq 2$, we use the generalisation of Supplementary Equation (97) to Hamiltonians with an arbitrary number of terms given in Eq. (3) of Ref. [13]. In particular,

$$U_O(t) = \left(\prod_{k=1}^{\Lambda} e^{-ic_1 h_k t}\right)\left(\prod_{k=\Lambda}^{1} e^{-id_1 h_k t}\right)\dots\left(\prod_{k=1}^{\Lambda} e^{-ic_4 h_k t}\right)\left(\prod_{k=\Lambda}^{1} e^{-id_4 h_k t}\right), \tag{99}$$

where $c_i = a_i - d_{i-1}$ (with $d_0 = 0$) and $d_i = b_i - c_i$.

1.3 THRIFT formulas

The circuit depths for implementing a THRIFT approximation of order p are the same as the corresponding Trotter approximation applied to the original Hamiltonian with the rearrangement

$$H = H_0 + \sum_{j=1}^{K}[(H_0 + \alpha h_j) - H_0] = H_0 + \sum_{j=1}^{K}(h'_j - H_0), \tag{100}$$

where we assume that the exponential of each $h'_j := H_0 + \alpha h_j$ can be implemented with $\mathcal{N}'_j = \mathcal{N}'$ arbitrary 2-qubit gates. Note that particular care is required in cases where H_0 contains terms acting on more that one qubit, as in the 1D Fermi-Hubbard model case discussed in Supplementary Section 2.4.

1.4 Magnus-THRIFT formulas

The first-order Magnus-THRIFT formula is given by Eq. (14) in the main text with $k = 1$,

$$\mathcal{S}_1^{\text{Magnus}}(t) = e^{-iH_0 t} e^{\Omega^{[1]}(t)}, \tag{101}$$

with

$$\Omega^{[1]}(t) = -i\int_0^t \tilde{H}_1(t_1)dt_1, \tag{102}$$

where $\tilde{H}_1(t) = e^{iH_0 t} H_1 e^{-H_0 t}$. In general, we can write $\tilde{H}_1(t) = \sum_{j=1}^{P} f_j(t)\tilde{h}_j$, where the exponential of each $\tilde{h}_j$ can be implemented with $\tilde{\mathcal{N}}_j$ layers of arbitrary 2-qubit gates. Hence

$$\int_0^t \tilde{H}_1(t_1)dt_1 = \sum_{j=1}^{P}\left(\int_0^t f_j(t_1)dt_1\right)\tilde{h}_j = \sum_{j=1}^{P} F_j(t)\tilde{h}_j = H_1^{\text{Magnus}}(t) \tag{103}$$

and

$$\mathcal{S}_1^{\text{Magnus}}(t) = e^{-iH_0 t} e^{-iH_1^{\text{Magnus}}(t)}. \tag{104}$$

Approximating the last term by a first-order Trotter formula, Supplementary Equation (104) can be implemented with $\sum_{j=1}^{P} \tilde{\mathcal{N}}_j$ layers of arbitrary 2-qubit gates.

The second-order Magnus-THRIFT formula is given by Eq. (14) in the main text with $k = 2$,

$$\mathcal{S}_2^{\text{Magnus}}(t) = e^{-iH_0 t} e^{\Omega^{[2]}(t)}, \tag{105}$$

with

$$\Omega^{[2]}(t) = -i\int_0^t \tilde{H}_1(t_1)dt_1 - \frac{1}{2}\int_0^t dt_1 \int_0^{t_1} dt_2 [\tilde{H}_1(t_1), \tilde{H}_1(t_2)]. \tag{106}$$

In this case, we can write $[\tilde{H}_1(t_1), \tilde{H}_1(t_2)] = \sum_{i,j=1}^{P} f_i(t_1) f_j(t_2)\tilde{h}_i\tilde{h}_j$, and therefore Eq. (106) becomes

$$\Omega^{[2]}(t) = -i\sum_{j=1}^{P'} g_j(t)\bar{h}_j, \tag{107}$$

with $P' \leq P^2$. The time-evolution operator of each term $\bar{h}_j$ can be implemented with arbitrary 2-qubit gate depth $\bar{\mathcal{N}}_j$. Approximating $e^{\Omega^{[2]}(t)}$ by a second-order Trotter formula and assuming for simplicity that $\bar{\mathcal{N}}_j = \bar{\mathcal{N}}$ for all j, we find that a single Trotter layer of Supplementary Equation (105) can be implemented with arbitrary 2-qubit gate depth $(2P' - 1)\bar{\mathcal{N}}$ and $N > 1$ Trotter layers with arbitrary 2-qubit gate depth $[(2P' - 2)N + 1]\bar{\mathcal{N}}$. Note that, in general, the terms $\bar{h}_j$ may contain multi-qubit terms and therefore $\bar{\mathcal{N}}$ depends on the specific model. In the next section we discuss the case of the 1D transverse-field Ising model.

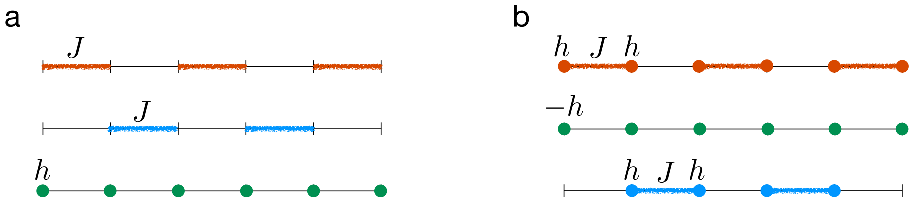

Supplementary Figure 1: Partitions for implementing the first-order (a) Trotter and (b) THRIFT formulas in the 1D TFIM and 1D Heisenberg models. (a) One layer of the first-order Trotter approximation for these models is obtained by approximating e^{-iHt} as $e^{-ih_1^{\text{odd}}}e^{-ih_1^{\text{even}}t}e^{-iH_0t}$. (b) One layer of the first-order THRIFT approximation is obtained by approximating e^{-iHt} as $e^{-i(h_1^{\text{odd}}+H_0)t}e^{iH_0t}e^{-i(h_1^{\text{even}}+H_0)t}$. Here, $h_1^{\text{even/odd}} = h_{XX}^{\text{even/odd}}$ for the 1D transverse-field Ising model and $h_1^{\text{even/odd}} = h_{XX+YY+ZZ}^{\text{even/odd}}$ for the 1D Heisenberg model. In each row of both panels, all the single-qubit (dots) and 2-qubit (thick lines) terms are implemented simultaneously.

2 Applications to specific models

2.1 1D transverse-field Ising model

For the 1D transverse-field Ising model, we have $H_{\text{TFIM}} = H_0 + JH_1$ with H_0 acting on single qubits only and $H_1 = h_{XX}^{\text{even}} + h_{XX}^{\text{odd}}$ (hence $K = P = 2$), with $h_{XX}^{\text{even/odd}} = \sum_{j\ \text{even/odd}} X_jX_{j+1}$. The time-evolution operators associated with such terms can be implemented with $\mathcal{N} = 1$ layer of arbitrary 2-qubit gates (or two layers of CNOT gates) each. Since the cost to implement the time-evolution operator of $h_{XX}^{\text{even/odd}} + H_0$ is the same as $h_{XX}^{\text{even/odd}}$ in terms of both arbitrary 2-qubit gates and CNOT gates, the circuit depth for a pth-order THRIFT formula is the same as for the corresponding Trotter formula.

For Omelyan et al.'s optimised small A formula, we have $\Lambda = 3$, and we identify $h_1 = H_0$, $h_2 = \alpha h_{XX}^{\text{even}}$, and $h_3 = \alpha h_{XX}^{\text{odd}}$. Recalling that H_0 can be implemented via single-qubit gates and merging the last exponential inside a bracket in Supplementary Equation (99) with the first exponential of the following one, we find that the arbitrary 2-qubit gate cost to implement N layers of Supplementary Equation (99) is $12N$ (corresponding to $24N$ layers of CNOT gates).

For Magnus-THRIFT 1 we have

$$\tilde{H}_1(t) = f_{XX}(t)H_{XX} + f_{YY}(t)H_{YY} + f_{XY+YX}(t)H_{XY+YX}, \tag{108}$$

with $H_{XX} = \sum_j X_jX_{j+1}$, $H_{YY} = \sum_j Y_jY_{j+1}$, $H_{XY+YX} = \sum_j (X_jY_{j+1} + Y_jX_{j+1})$, and $f_i(t)$ time-dependent coefficients. Similarly to the previous cases, $\tilde{H}_1(t)$ can be split into even/odd contributions, each of which can be implemented with one layer of arbitrary 2-qubit gates (or two CNOT gates). Hence, we have $P = 2$ and $\tilde{\mathcal{N}}_j = \tilde{\mathcal{N}} = 1$: $N > 1$ layers of the Magnus-THRIFT 1 formula in Supplementary Equation (104) can be implemented with $2N$ layers of arbitrary 2-qubit gates (or $4N$ layers of CNOT gates). For Magnus-THRIFT 2, Supplementary Equation (106) can be written as

$$\begin{aligned}\Omega^{[2]}(t) \propto\ & f_{XX}(t)H_{XX} + f_{YY}(t)H_{YY} + f_{XY+YX}(t)H_{XY+YX} \\ & + f_{XZY+YZX}(t)H_{XZY+YZX} + f_{XZX}(t)H_{XZX} + f_{YZY}(t)H_{YZY} \\ & + \text{single-qubit terms.}\end{aligned} \tag{109}$$

Here, $H_{XZY+YZX} = \sum_j X_jZ_{j+1}Y_{j+2} + Y_jZ_{j+1}X_{j+2}$, $H_{XZX} = \sum_j X_jZ_{j+1}X_{j+2}$, $H_{YZY} = \sum_j Y_jZ_{j+1}Y_{j+2}$, and the various $f_i(t)$ denote the corresponding time-dependent coefficients. Since the terms in the second line of Supplementary Equation (109) act on three qubits, $\Omega^{[2]}(t)$ has to be split into three groups as shown in Supplementary Figure 2(a). Moreover, one can show numerically that the time-evolution operator of each group can be implemented with 3 layers of arbitrary 2-qubit gates (corresponding to 9 layers of CNOT gates). Hence, the second-order Magnus-THRIFT formula for the 1D transverse-field Ising model one has $P' = 3$ and $\tilde{\mathcal{N}}_j = \tilde{\mathcal{N}} = 3$, corresponding to an arbitrary 2-qubit gate depth of $12N + 3$ (and CNOT gate depth of $36N + 9$).

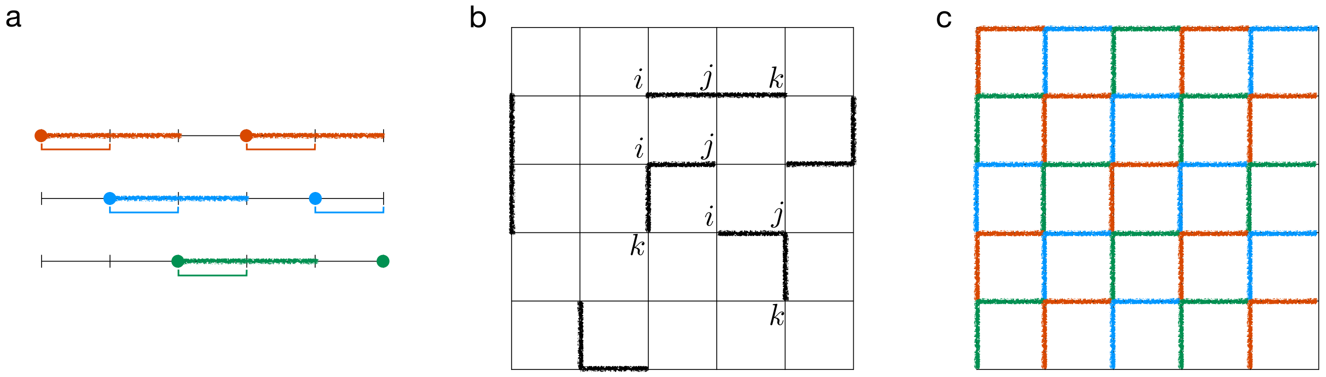

Supplementary Figure 2: Partitions for implementing the second-order Magnus-THRIFT formula in the 1D (a) and 2D (b,c) transverse-field Ising model. (a) One of the possible groupings of the terms in $\Omega^{[2]}(t)$ in Supplementary Equation (109). In each one of the three subgroups, all the single-qubit (dots), 2-qubit (thin lines), and three-qubit (thick lines) terms are implemented simultaneously. (b) In the 2D transverse-field Ising model, there are six possible configurations for each of the three-qubit terms in $\Omega^{[2]}(t)$ in Supplementary Equation (109). (c) In order to implement $e^{\Omega^{[2]}(t)}$, each of the six possible configurations can be split into three layers of terms that can be implemented simultaneously.

2.2 2D transverse-field Ising model

For the 2D transverse-field Ising model we have $H_{\mathrm{TFIM}} = H_0 + JH_1$, with $H_1 = H^{\mathrm{h}}_{XX} + H^{\mathrm{v}}_{XX} = h^{\mathrm{h,even}}_{XX} + h^{\mathrm{h,odd}}_{XX} + h^{\mathrm{v,even}}_{XX} + h^{\mathrm{v,odd}}_{XX}$, with the h and v superscripts denoting horizontal and vertical terms, respectively. Hence, we have $K = 4$. The time-evolution operator corresponding to each term can be implemented with a layer of $\mathcal{N} = 1$ arbitrary 2-qubit gates (or 2 CNOT gates). Similarly to the 1D case, one can implement the time-evolution operators of $h^{\mathrm{h/v,even/odd}}_{XX} + H_0$ occurring in THRIFT formulas with the same cost.

For Omelyan et al.'s optimised small A formula we have $\Lambda = 5$, with $h_1 = H_0$, $h_2 = \alpha h^{\mathrm{h,even}}_{XX}$, $h_3 = \alpha h^{\mathrm{h,odd}}_{XX}$, $h_4 = \alpha h^{\mathrm{v,even}}_{XX}$, and $h_5 = \alpha h^{\mathrm{v,odd}}_{XX}$ (see Supplementary Section 2.2). With similar arguments as in the 1D case, we find that the arbitrary 2-qubit gate cost to implement N layers of Supplementary Equation (99) is $28N$ (or $56N$ layers of CNOT gates).

In Magnus-THRIFT 1, $\tilde{H}_1(t)$ has the same form as Supplementary Equation (108) and can be split into four terms as the original H_1. Therefore, we find $P = 4$ and $\tilde{\mathcal{N}}_j = \tilde{\mathcal{N}} = 1$: $N > 1$ layers of the Magnus-THRIFT 1 formula in Supplementary Equation (104) can be implemented with $4N$ layers of arbitrary 2-qubit gates (or $8N$ layers of CNOT gates).

The implementation of the second-order Magnus-THRIFT approximation requires more care. The functional form of $\Omega^{[2]}(t)$ is the same as in Supplementary Equation (109), but each of the three-qubit Hamiltonians $H_{XZY+YZX}$, H_{XZX}, and H_{YZY} has now a 2D nature. For instance, $H_{XZX} = \sum_{\langle i,j\rangle} \sum_{k\in \mathrm{neigh}(\{i,j\})} X_i Z_j X_k$: here, i, j are nearest-neighbors and k is a nearest-neighbor of either i or j. Hence, for a given choice of i, j, there are 2 linear (vertical and horizontal) and 4 two-dimensional "L"-shaped independent configurations (see Supplementary Figure 2(b)). The time-evolution operators corresponding to each of these terms can be implemented in 3 layers as shown in Supplementary Figure 2(c). Then, $P' = 18$. In turn, we numercally verified that each layer can be implemented with $\bar{\mathcal{N}} = 3$ arbitrary-two qubit gates (or 9 CNOT gates). The overall arbitrary 2-qubit (CNOT) gate depth to implement $N > 1$ steps is therefore $102N + 3$ $(306N + 9)$.

2.3 1D Heisenberg model

Similarly to the 1D transverse-field Ising model, for the 1D Heisenberg model we have $H_{\mathrm{Heisenberg}} = H_0 + JH_1$ with H_0 acting on single qubits only and $H_1 = H_{XX} + H_{YY} + H_{ZZ} = h^{\mathrm{even}}_{XX+YY+ZZ} + h^{\mathrm{odd}}_{XX+YY+ZZ}$ (hence $K = 2$), with $h^{\mathrm{even/odd}}_{XX+YY+ZZ} = \sum_{j\ \mathrm{even/odd}} (X_j X_{j+1} + Y_j Y_{j+1} + Z_j Z_{j+1})$. Therefore, the circuit depths for Trotter and THRIFT formulas for the 1D Heisenberg model can be obtained by following the same steps as the 1D transverse-field Ising model discussed in Supplementary Section 2.1. The same holds for Omelyan et al.'s optimised small A formula,

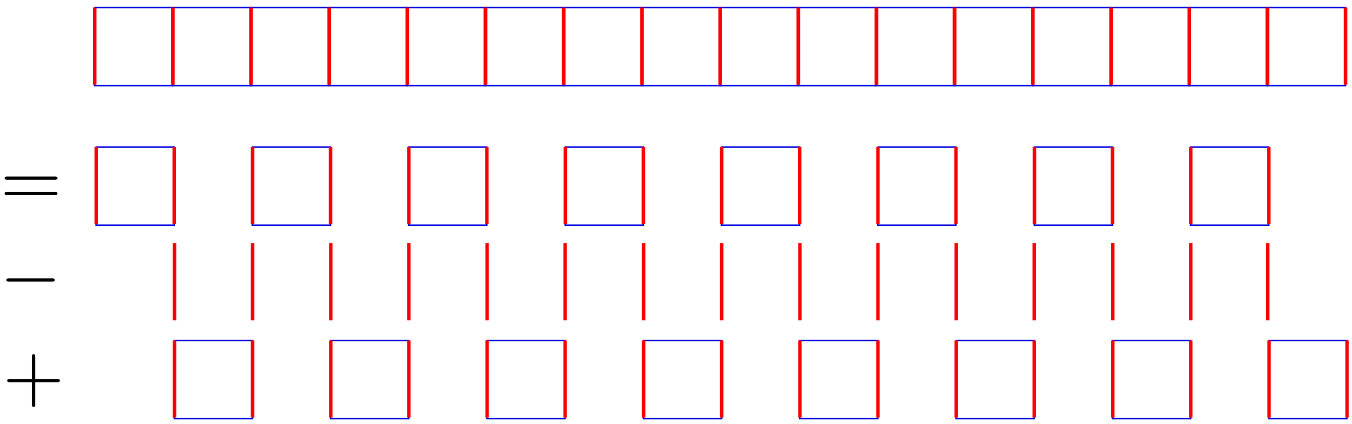

Supplementary Figure 3: Partition of terms for THRIFT in the 1D Fermi-Hubbard model. Vertical lines (red) correspond to the on-site interaction, while horizontal lines (blue) correspond to the hopping terms. Taking $H_0 = \sum_j n_{j\uparrow}n_{j\downarrow}$ leads to a partition where 4 qubit gates are needed.

where $\Lambda = 3$ and we identify $h_1 = H_0$, $h_2 = \alpha h^{\text{even}}_{XX+YY+ZZ}$, and $h_3 = \alpha h^{\text{odd}}_{XX+YY+ZZ}$. In particular, the arbitrary 2-qubit gate depths for the various formulas are the same, while to obtain the CNOT gate depths, one has to take into account that the time-evolution operator associated with $h^{\text{even/odd}}_{XX+YY+ZZ}$ uses 3 layers of CNOT gates.

2.4 1D Fermi-Hubbard model

The Hamiltonian of the Fermi-Hubbard model can be written as $H_{\text{FH}} = H_0 + t_{\text{hop}}H_1$, with $H_0 = H_{\text{int}}$ and $H_1 = H_{\text{hop}} = h^{\text{even}}_{\text{hop}} + h^{\text{odd}}_{\text{hop}}$, with $h^{\text{even/odd}}_{\text{hop}} = -\sum_\sigma \sum_{i\ \text{even/odd}} \left(c^\dagger_{i,\sigma} c_{i+1,\sigma} + c^\dagger_{i+1,\sigma} c_{i,\sigma} \right)$. The time-evolution operator corresponding to each term of this decomposition can be implemented with one layer of arbitrary 2-qubit gates (and 2 layers of CNOT gates). Hence, we find $K = 3$ and $\mathcal{N} = 1$.

For Omelyan et al.'s optimised small A formula we have $\Lambda = 3$ with $h_1 = H_{\text{int}}$, $h_2 = \alpha h^{\text{even}}_{\text{hop}}$, and $h_3 = \alpha h^{\text{odd}}_{\text{hop}}$ (see Supplementary Section 2.4). In contrast to the previous cases, here H_0 can be implemented with one layer of arbitrary 2-qubit gates. Hence, the overall arbitrary 2-qubit gate cost to implement N layers of Supplementary Equation (99) is $16N + 1$ (corresponding to $32N + 2$ layers of CNOT gates). Note that, to obtain this number, one needs to merge the last term $e^{-id_4 H_{\text{int}} t}$ of the ith Trotter layer with the first term $e^{-ic_1 H_{\text{int}} t}$ of the $(i+1)$st layer.

Finally, there are some additional considerations to obtain the 2-qubit gate depth for THRIFT formulas. In this case, H_0 is not a single-qubit Hamiltonian and implementing $e^{\pm iH_0 t}$ requires $\mathcal{N}'_0 = 1$ layer of arbitrary 2-qubit gates (corresponding to 2 layers of CNOT gates). As shown in Supplementary Figure 3, each of the $K = 2$ THRIFT partitions $[H_0 + H^{\text{even/odd}}]$ consist of terms acting on four qubits and implementing $e^{-i(H_0 + H^{\text{even/odd}})t}$ requires 3 layers of arbitrary 2-qubit gates (and 6 layers of CNOT gates). Hence, $\mathcal{N}'_1 = 3$. The overall arbitrary 2-qubit (CNOT) gate depth can be computed by taking into account both these facts. For instance, $N > 1$ layers of the second-order THRIFT formula can be implemented with arbitrary 2-qubit gate depth $[(2K-2)\mathcal{N}'_1 + 2\mathcal{N}'_0)]N + \mathcal{N}'_1 = 8N + 3$.

Supplementary Note 4 – Additional numerical results

In the Discussion section of the main text, we showed the 2-qubit gate depth d to achieve a fixed precision ϵ for different system sizes L at evolution time $T = L$. In this section we provide a more detailed analysis by showing the results at different values of the small parameter α, performing weighted linear regression to the power laws describing the depth d as a function of L, and comparing the power laws thus obtained to the theoretically expected results.

For later reference we note that for ordinary kth-order Trotter methods, the depth to achieve error ϵ scales for evolution time T in a system of size L scales as

$$d_{\text{trotter},k} = O\left(\epsilon^{-\frac{1}{k}} \alpha^{\frac{1}{k}} L^{\frac{1}{k}} T^{1+\frac{1}{k}}\right) \tag{110}$$

if we choose a splitting of the Hamiltonian that has H_0 as one term and all other terms scale linear with α. For a kth-order THRIFT formula, almost the same is true; the only difference is that the commutator bounds now give a factor of α^2, so the depth scales as

$$d_{\text{thrift},k} = O\Big(\epsilon^{-\frac{1}{k}}\alpha^{\frac{2}{k}}L^{\frac{1}{k}}T^{1+\frac{1}{k}}\Big). \tag{111}$$

These two expressions follow simply from combining the ordinary Trotter error bounds, or THRIFT error bounds given in Theorem 1 in the main text, with the analysis from Supplementary Section 1. Because we only consider geometrically local Hamiltonians, Supplementary Equation (110) and (111) hold with ϵ denoting the worst-case error, as in Figure 2 in the main text, as well as when ϵ is the average case error, as in Figures 5 and 12 in the main text, by the same analysis done for Theorem 2 in [2].

1 Transverse-field Ising model

In Supplementary Figure 4 we analyse in more detail the 2-qubit depth required to achive a worst-case error $\|U - U_{\text{exact}}\| < 0.01$ for the different TDS methods as a function of system size L and evolution time T (1D case) or only evolution time T (2D case). On the left we show the 2-qubit gate depth d at fixed $\alpha = 1/8$ and see that it is well described by a power law of the form $d = aL^k$ (1D) or $d = aT^k$ (2D). On the right we plot the prefactor a and exponent k as a function of α. In the 1D case, the exponents of the second- and fourth-order methods match the theoretically expected values of 2 and 1.5 very well. The same is true for the optimised eighth-order formula where the exponent is ≈ 1.125 for all α. The exponents of the first- and second-order methods match. This is because the transverse-field Ising model Hamiltonian and $\tilde{H}_1(t)$ both can be decomposed into only two terms that are exactly implementable, in which case the first-order Trotter formula has the same scaling as the second-order formula. The fit exponent of the optimised THRIFT 8 formula, on the other hand, does not match the theoretically expected value and is below 1 for all α, despite the very accurate fits shown on the left. Instead we find that a scales roughly as $\alpha^{\frac{2}{k}}$ for second-, fourth-, and eighth-order THRIFT *and* Trotter methods, although the prefactors a of the THRIFT methods are always below those of the corresponding Trotter method. Again, the first-order methods behave similarly to the second-order methods and a is roughly linear in a for both Magnus-THRIFT methods.

In the 2D case the fit exponents do not fall as nicely into distinct groups, but we observe again that, with the notable exception of Trotter 1, all first- and second-order methods have exponent $k \approx 1.5$ as theoretically expected for second-order methods. The fourth-order methods have $k \approx 1.25$, again in line with theoretical expectations. Trotter 1 and the optimised THRIFT 8 formula, on the other hand, deviate substantially from the theoretical expectation with $k \approx 1$ and $k \approx 0.5$, respectively. This suggests that the optimised THRIFT 8 formula can be used to fast forward the transverse-field Ising model. While the 1D transverse-field Ising model is integrable, this is more surprising in the 2D case and may be an artifact of the fairly small system size considered here. For the prefactors a, we find the same as in 1D: they have d scaling like $\alpha^{\frac{2}{k}}$ for THRIFT *and* Trotter methods, i.e., as theoretically expected for the THRIFT methods.

2 1D Heisenberg model

In Supplementary Figure 5 we analyse the 2-qubit gate depth to achieve an average infidelity $\mathbb{E}_{\{|x\rangle\}}[1-|\langle x|U^\dagger_{\text{exact}}U|x\rangle|^2] \leq 0.01$ as a function of the system size L, evolution time T, and interaction strength $J = \alpha$. On the left we show the 2-qubit gate depth at fixed $\alpha = \frac{1}{8}$, which is well described by a power law of the form $d = aL^k$. We find that this remains true for different choices of α, where the coefficients a and k depend on α. On the right we show the coefficients obtained via weighted linear regression as a function of α. While the situation is not as clear cut as for the transverse-field Ising model in Supplementary Figure 4, the algorithms (with maybe the exception of the optimised small A method) still appear to fall into two groups: the first- and second-order methods, for which (at least for larger $\alpha \gtrsim 0.2$) $k \approx 1.75$, and the higher-order methods, for which (again, at least for $\alpha \gtrsim 0.2$) $k \approx 1.25$.

3 1D Fermi-Hubbard model with weak hopping

In addition to the two spin-models simulated in the main text we also considered the 1D Fermi-Hubbard model with weak hopping as an example of a fermionic simulation. The Hamiltonian of the Fermi-Hubbard model is

$$H_{\text{FH}} = -t_{\text{hop}} \sum_{\langle i,j\rangle,\sigma} \Big(c^\dagger_{i,\sigma}c_{j,\sigma} + c^\dagger_{j,\sigma}c_{i,\sigma}\Big) + U\sum_i n_{i\uparrow}n_{i\downarrow}, \tag{112}$$

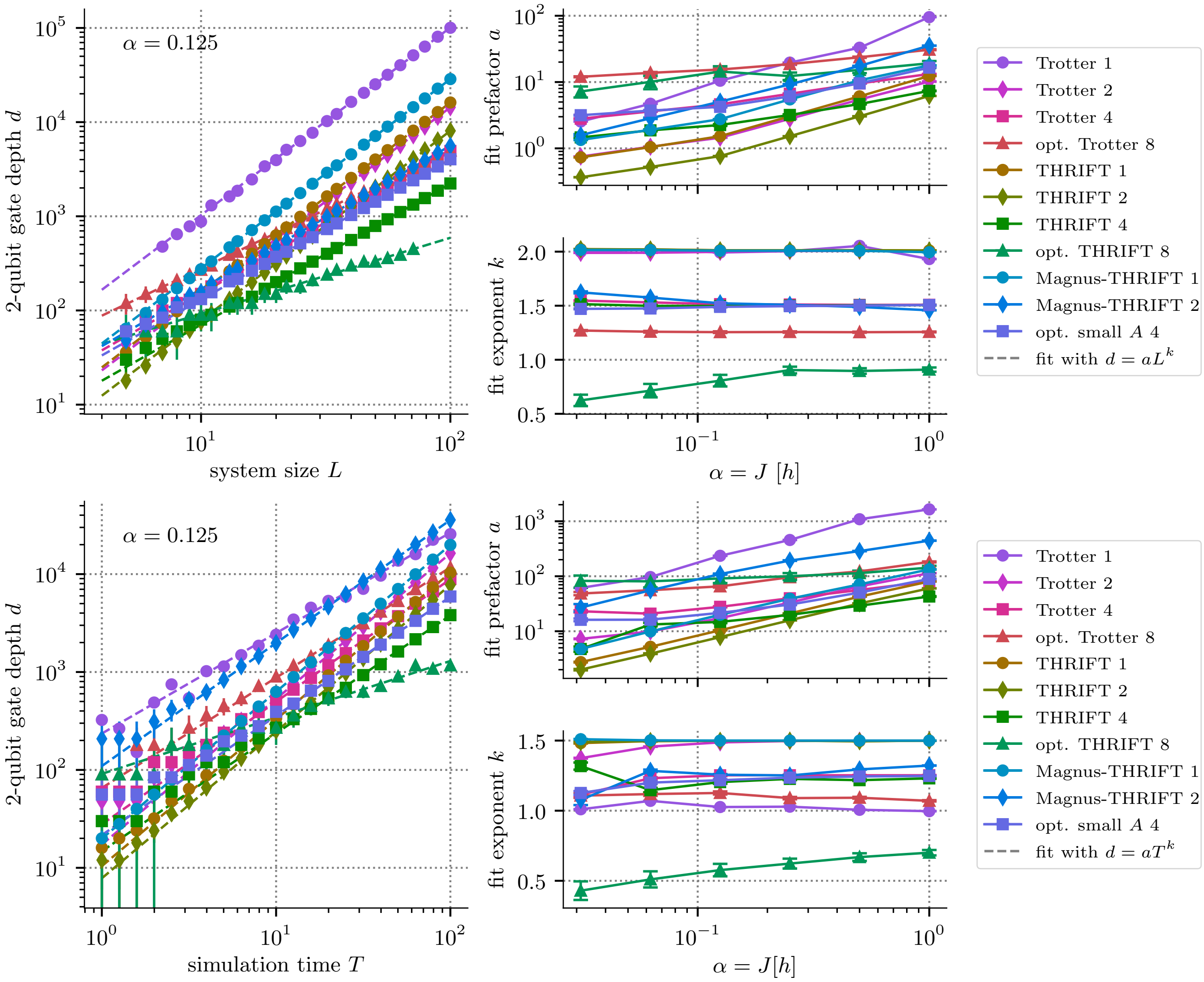

Supplementary Figure 4: Performance scaling for the 1D (top) and 2D (bottom) transverse-field Ising model. (left) The same data as in Figure 2 in the main text to provide context to the fit parameters shown on the right. (right) Fit parameters of a power law $d = aL^k$ or $d = aT^k$, respectively, to the data shown on the left for different values of α, obtained via weighted linear regression.

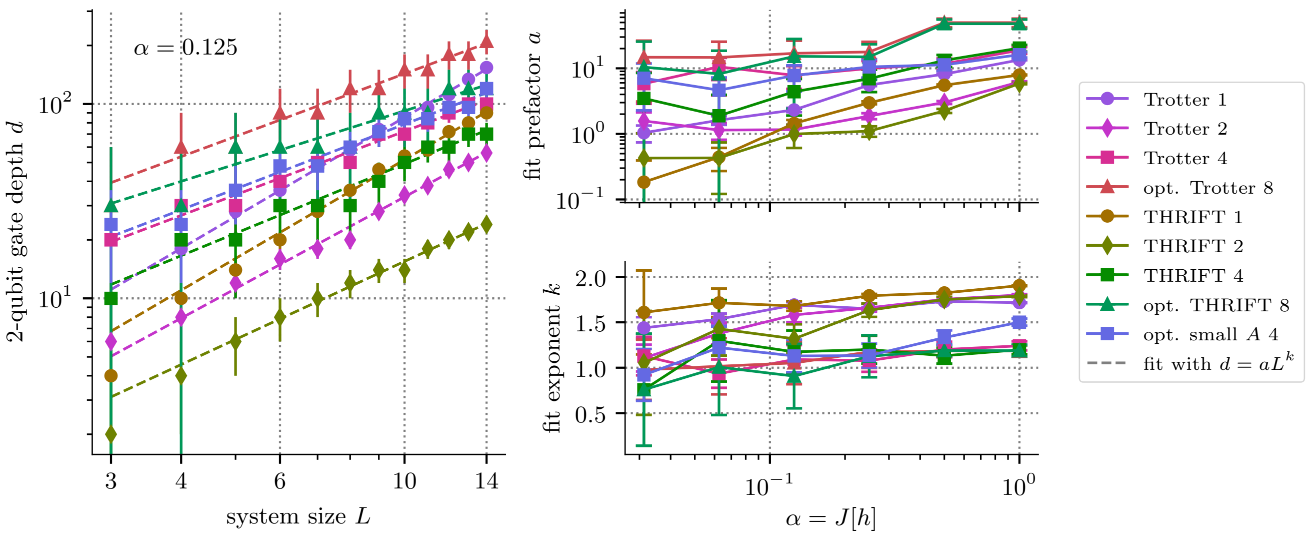

Supplementary Figure 5: Performance scaling for the 1D Heisenberg chain. (left) The same data as in Figure 5 in the main text to provide context for the fit parameters shown on the right. (right) Fit parameters for the data shown on the left for different values of α, obtained via weighted linear regression.

where $c_{i,\sigma}^{(\dagger)}$ are the fermionic annihilation (creation) operators on site i with spin σ and $n_{i,\sigma} = c_{i\sigma}^\dagger c_{i\sigma}$ are the corresponding number operators. The first sum runs over all edges $\langle i,j \rangle$ of the lattice and the second over all sites i.

In numerical simulations, we fix the interaction strength $U = 1$, let $\alpha := -t_{\text{hop}}$ be the small parameter, and measure time T in units of U^{-1}. To map the fermionic Hamiltonian to qubits, we use the Jordan-Wigner transformation and the same circuits developed in [14]. This results in a ladder-like interaction graph of the qubit Hamiltonian with one rung corresponding to the spin-up state on a site and one to the spin-down state on that site. As discussed in Supplementary Section 2.4, the interaction Hamiltonian $\tilde{H}_1(t)$ then consists of terms acting on four qubits, corresponding to the spin-up and spin-down states on neighbouring sites (see Supplementary Figure 3). We find numerically that time evolution with such a four-local term can be decomposed into a product of three evolutions with the hopping and three evolutions with the interaction terms for all values of T/N (the Trotter time step), t_{hop}, and U. This means that one step of any THRIFT circuit takes three times the 2-qubit gate depth of the corresponding Trotter circuit. This is in contrast to the transverse-field Ising model or Heisenberg model where the depth of THRIFT and Trotter methods is the same, because H_0 is 1-local and hence $\tilde{H}_1(t)$ has the same locality as H_1. The 2-qubit gate depths per step and number of steps used in Supplementary Figure 6 are shown in Supplementary Table 2.

In Supplementary Figures 6 and 7 we repeat, for 1D Fermi-Hubbard chains, the same numerical analysis that we did for the transverse-field Ising model in Figures 1 and 2 in the main text and for the 1D Heisenberg model in Figures 4 and 5 in the main text. Because the Fermi-Hubbard model needs two qubits per site—one for each spin direction—and is not integrable, we are limited to much smaller system sizes, and for the depth scaling shown in Supplementary Figure 7, we again use the average infidelity $\mathbb{E}_{|x\rangle}[1 - |\langle x|U_{\text{exact}}^\dagger U|x\rangle|^2]$ instead of the more costly worst-case error $\|U_{\text{exact}} - U\|$. We find that within the range of T and $\alpha = -t_{\text{hop}}$ that we study, THRIFT methods rarely outperform ordinary Trotter methods, and in the regions they do (i.e., for $\alpha \leq 10^{-2}$) they are beaten by the "small A" method of Omelyan et al. In particular, the optimised eighth-order Trotter formula of [11] and the "small A" method of Omelyan et al. perform best out of all tested formulas for a wide range of T and α. The main reason for the poor performance of THRIFT can be traced back to the high cost of implementing the gates arising in the THRIFT decomposition, as can be seen in Supplementary Table 2. Indeed, as explained in detail in Supplementary Section 2.4, the latter contains 4-local terms that are each implemented with 3 layers of arbitrary 2-qubit gates. Similar conclusions can be drawn by looking at the 2-qubit gate depths required to achieve a fixed average infidelity as a function of system size L and evolution time T, as shown in Supplementary Figure 7. Even for $\alpha = 1/16$, the Trotter methods have lower circuit depths than the corresponding THRIFT methods. The scaling exponents with L and T broadly agree with those expected from the theory results in Supplementary Sections 1 and 2 and are analysed

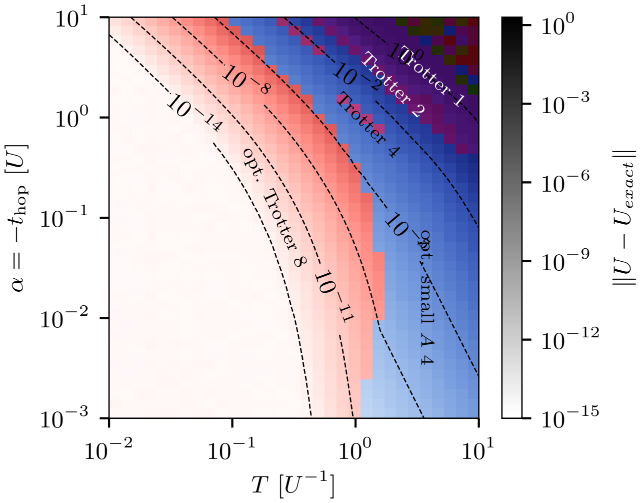

Supplementary Figure 6: Landscape of the best TDS algorithm, as measured by the worst-case error $\|U - U_{\mathrm{exact}}\|$, as a function of the hopping strength t_{hop} and evolution time T at identical circuit depth for a 1×5 Fermi-Hubbard chain. The circuit depth is fixed to 1 step of THRIFT 4 evolution. For the other algorithms, the number of steps is chosen to match the 2-qubit depth as closely as possible according to the 2-qubit depths shown in Supplementary Table 2. The colour of each point represents the algorithm that achieves the lowest error at those values of t_{hop} and T, while the brightness indicates the magnitude of the error.

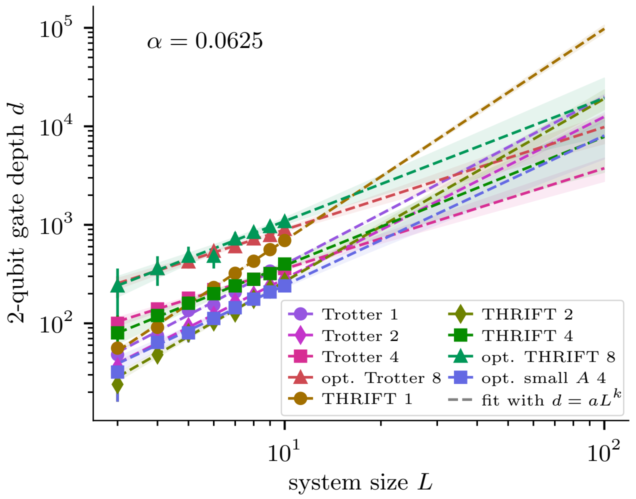

Supplementary Figure 7: 2-qubit depth to achieve average infidelity $\mathbb{E}_{\{|x\rangle\}}[1 - |\langle x|U_{\mathrm{exact}}^{\dagger} U|x\rangle|^2] \leq 0.01$ for the different TDS algorithms for a $1 \times L$ Fermi-Hubbard chain with an interaction strength of $t_{\mathrm{hop}} = 1/16$ and evolution time $T = 2L$. Unlike Figure 2 in the main text, we use average fidelity to be able to simulate larger system sizes. Error bars are ± 1 step and the shaded regions are the one-sigma confidence intervals of the extrapolations. Note that, unlike the transverse-field Ising model and Heisenberg models, the extrapolation from small sizes is not as conclusive in this case, making it difficult to determine the best-performing algorithm in the 100-qubit regime.

Algorithm	2-qubit gate depth	CNOT depth	# steps in Supplementary Figure 6
Trotter 1	$3N$	$6N$	20
Trotter 2	$4N+1$	$8N+2$	15
Trotter 4	$20N+1$	$40N+2$	3
optimised Trotter 8	$60N+1$	$120N+2$	1
THRIFT 1	$7N$	$14N$	8
THRIFT 2	$8N+3$	$16N+6$	7
THRIFT 4	$40N+3$	$80N+6$	1
optimised THRIFT 8	$120N+3$	$240N+6$	N/A
optimised small A 4	$16N+1$	$32N+2$	3

Supplementary Table 2: Circuit depth comparison of the different TDS algorithms investigated and shown in Supplementary Figure 6 for the 1D Fermi-Hubbard model. The first column shows the 2-qubit depth of the circuit corresponding to N Trotter steps in terms of arbitrary 2-qubit gates. The second column shows the corresponding cost in terms of CNOT gates. Finally, the third column gives the number of Trotter steps used in Supplementary Figure 6, which correspond to a fixed budget of arbitrary 2-qubit gates of 61. Note that, in the latter, we do not include the optimised THRIFT 8 algorithm since a single step requires deeper circuits than we allowed for Supplementary Figure 6 and increasing the circuit depth would result in most of Supplementary Figure 6 being dominated by the numerical noise floor.

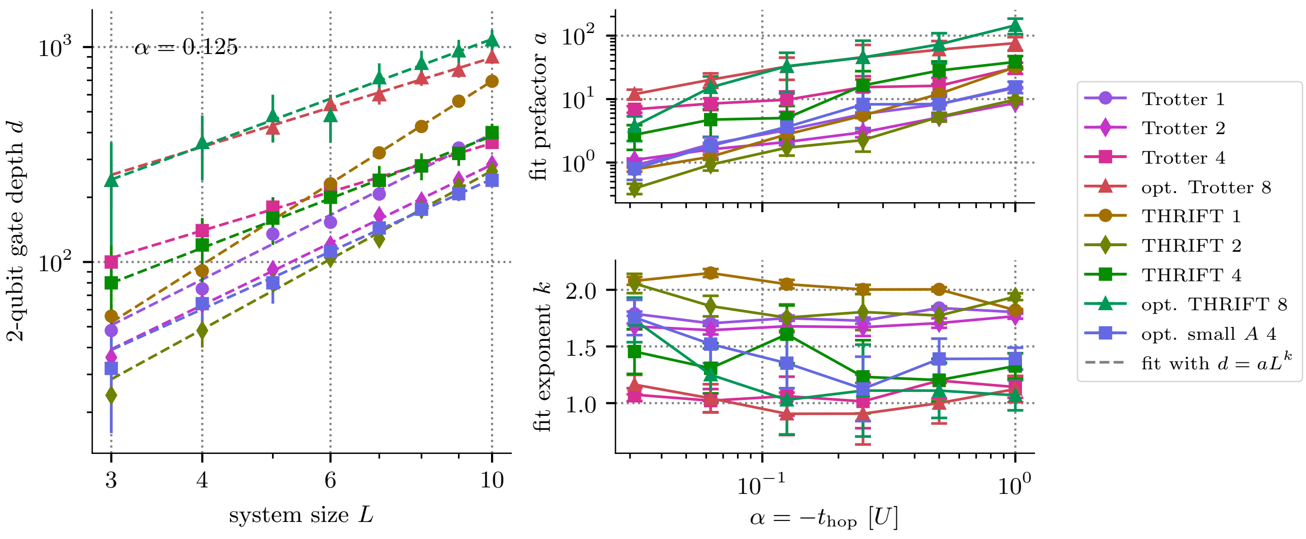

Supplementary Figure 8: Performance scaling for the 1D Fermi-Hubbard model. (left) The same data as in Supplementary Figure 7 to provide context for the fit parameters shown on the right. (right) Fit parameters to the data shown on the left for different values of α. The error bars are the fit uncertainties when taking the error bars from the left as the uncertainties of the original data.

in more detail and as a function of α in Supplementary Figure 8.

Given the data shown in Supplementary Figure 6, we chose not to numerically study the performance of the Magnus-THRIFT algorithms for the Fermi-Hubbard model. Since THRIFT methods only become advantageous for $\alpha \leq 10^{-2}$ with respect to Trotter methods due to the more complex gates needed for the THRIFT circuits, and the second-order Magnus-THRIFT Hamiltonian $\Omega^{(2)}$ has up to 6-local terms that must be split into at least three simultaneously implementable terms (assuming the ability to implement arbitrary 6-qubit gates), we expect that the values of α for which Magnus-THRIFT becomes advantageous are rather small.

The same analysis done for the transverse-field Ising model and Heisenberg model in Supplementary Figures 4 and 5 is repeated for the Fermi-Hubbard model in Supplementary Figure 8. Again, we use the average infidelity $\mathbb{E}_{\{|x\rangle\}}[1 - |\langle x|U_{\text{exact}}^\dagger U|x\rangle|^2] \leq 0.01$ as a figure of merit to be able to reach larger system sizes in our simulations. Again, we find robust power laws for the 2-qubit depth to get the average infidelity below threshold as we increase the system size L and scale the evolution time as $T = L$, as exemplified for $\alpha = 1/16$ in the left of Supplementary Figure 8. On the right we plot the exponents k and prefactors a of that power law as a function of α.

As in the case of Heisenberg model (Supplementary Figure 5), the algorithms do not fall as neatly into groups with different exponents as for the transverse-field Ising model (Supplementary Figure 4). Trotter 1 and 2 have $k \approx 1.75$ for all α, and Trotter 4 and the optimised Trotter 8 formula have $1 \lesssim k \lesssim 1.25$ for all α, but also fairly large uncertainties. For THRIFT 1 and 2, k varies between 2.25 and 1.75, and for THRIFT 4 and 8, it decreases with α from $k \approx 1.75$ at $\alpha = 1/32$ to $k \approx 1.25$ at $\alpha = 1$.

4 TFIM and Heisenberg model with strong interactions $\alpha = 1$

Figures 1 and 4 in the main text indicate that the THRIFT methods perform well for the transverse-field Ising model and Heisenberg model not only in the theoretically expected $\alpha \ll 1$ limit, but also for $\alpha \sim 1$. In Supplementary Figures 9 and 10 we show that this is indeed the case by repeating the numerics done for $\alpha = 1/8$ in Figures 2 and 5 in the main text, now taking the larger value $\alpha = 1$. We find that for the transverse-field Ising model, the THRIFT circuits use lower depth than Trotter circuits to achieve a desired precision even at $J = h$, and that for the Heisenberg model, the depths are very similar for the THRIFT and Trotter methods.

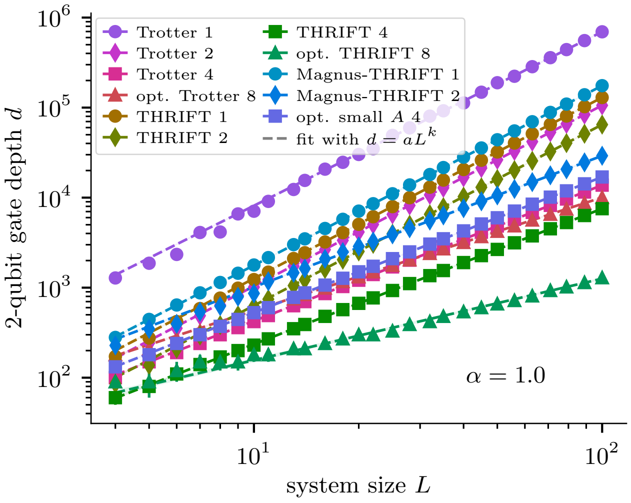

Supplementary Figure 9: 2-qubit gate depth to achieve $\|U - U_{\text{exact}}\| \leq 0.01$ for the different TDS algorithms for a field strength of $J = 1$ and evolution time $T = L$, for a $1 \times L$ Ising chain with transverse field $h = 1$. In contrast to Figure 2 in the main text, we have $\alpha = 1$, soTheorem 1 in the main text does not predict that THRIFT methods should outperform Trotter methods. Nevertheless, THRIFT uses shallower circuits to achieve the desired precision than the corresponding Trotter methods.

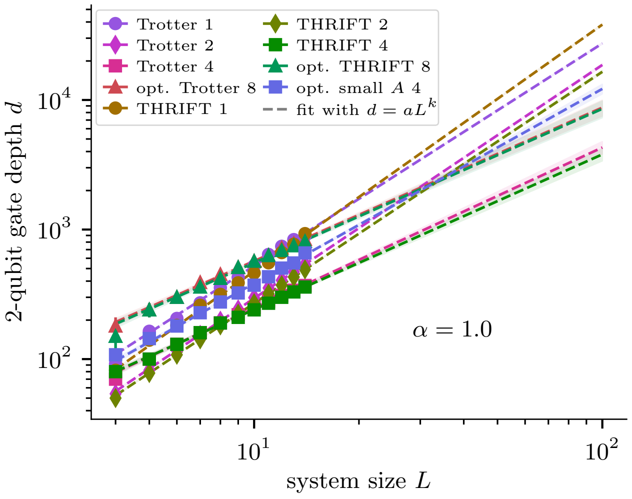

Supplementary Figure 10: 2-qubit depth to achieve average infidelity $\mathbb{E}_{\{|x\rangle\}}[1 - |\langle x|U^\dagger_{\text{exact}}U|x\rangle|^2] \leq 0.01$ for the different TDS algorithms for a $1 \times L$ Heisenberg chain with field strength of $J = 1$ and evolution time $T = L$. In contrast to Figure 5 in the main text, we have $\alpha = 1$ here, so Theorem 1 in the main text does not predict that THRIFT methods should outperform Trotter methods. Nevertheless, THRIFT uses almost the same circuit depth as the corresponding Trotter methods to achieve the target precision.

Supplementary References

[1] A. M. Childs, Y. Su, M. C. Tran, N. Wiebe, and S. Zhu, "Theory of Trotter error with commutator scaling", Phys. Rev. X **11**, 011020 (2021).

[2] Q. Zhao, Y. Zhou, A. F. Shaw, T. Li, and A. M. Childs, "Hamiltonian simulation with random inputs", Phys. Rev. Lett. **129**, 270502 (2022).

[3] Etienne (https://math.stackexchange.com/users/80469/etienne), *Assume that* $f \in L([a, b])$ *and* $\int x^n f(x)dx = 0$ *for* $n = 0, 1, 2 \ldots$ Mathematics Stack Exchange, URL:https://math.stackexchange.com/q/876642 (version: 2014-07-24), eprint: `https://math.stackexchange.com/q/876642`.

[4] P. C. Moan, *Efficient approximation of Sturm-Liouville problems using Lie-group methods*, Numerical Analysis Reports (University of Cambridge, Department of Applied Mathematics and Theoretical Physics, 1998).

[5] E. T. Bell, "Exponential polynomials", Ann. Math. **35**, 258–277 (1934).

[6] L. Comtet, *Advanced combinatorics: the art of finite and infinite expansions* (Springer Netherlands, 1974).

[7] A. D. D. Craik, "Prehistory of Faà di Bruno's formula", Am. Math. Mon. **112**, 119–130 (2005).

[8] F. Fer, "Résolution de l'équation matricielle du/dt = pu par produit infini d'exponentielles matricielles", Bull. Cl. Sci., Acad. R. Belg. **44**, 818–829 (1958).

[9] A. Iserles, "Solving linear ordinary differential equations by exponentials of iterated commutators", Numer. Math. **45**, 183–199 (1984).

[10] N. Hatano and M. Suzuki, "Finding exponential product formulas of higher orders", in *Quantum annealing and other optimization methods* (Springer Berlin, Heidelberg, 2005), pp. 37–68.

[11] M. E. S. Morales, P. C. S. Costa, D. K. Burgarth, Y. R. Sanders, and D. W. Berry, *Greatly improved higher-order product formulae for quantum simulation*, 2022, arXiv:2210.15817.

[12] I. Omelyan, I. Mryglod, and R. Folk, "Optimized Forest–Ruth- and Suzuki-like algorithms for integration of motion in many-body systems", Comput. Phys. Commun. **146**, 188–202 (2002).

[13] J. Ostmeyer, "Optimised Trotter decompositions for classical and quantum computing", J. Phys. A **56**, 285303 (2023).

[14] C. Cade, L. Mineh, A. Montanaro, and S. Stanisic, "Strategies for solving the Fermi-Hubbard model on near-term quantum computers", Phys. Rev. B **102**, 235122 (2020).
